# Selenium and Selenoproteins in Adipose Tissue Physiology and Obesity

**DOI:** 10.3390/biom10040658

**Published:** 2020-04-24

**Authors:** Alexey A. Tinkov, Olga P. Ajsuvakova, Tommaso Filippini, Ji-Chang Zhou, Xin Gen Lei, Eugenia R. Gatiatulina, Bernhard Michalke, Margarita G. Skalnaya, Marco Vinceti, Michael Aschner, Anatoly V. Skalny

**Affiliations:** 1 Yaroslavl State University, 150003 Yaroslavl, Russia; oajsuvakova@gmail.com (O.P.A.);; 2 IM Sechenov First Moscow State Medical University (Sechenov University), 119146 Moscow, Russia; 3 Federal Research Centre of Biological Systems and Agro-Technologies of the Russian Academy of Sciences, 460000 Orenburg, Russia; 4 CREAGEN, Environmental, Genetic and Nutritional Epidemiology Research Center, University of Modena and Reggio Emilia, 41121 Modena, Italy; 5 School of Public Health (Shenzhen), Sun Yat-sen University, Shenzhen 518100, China; 6 Department of Animal Science, Cornell University, Ithaca, NY 14853, USA; 7 All-Russian Research Institute of Medicinal and Aromatic Plants (VILAR), 117216 Moscow, Russia; 8 Helmholtz Zentrum München, Ingolstädter Landstraße 1, 85764 Neuherberg, Germany; 9 Department of Molecular Pharmacology, Albert Einstein College of Medicine, Bronx, NY 10461, USA

**Keywords:** selenium, selenoprotein, adipocyte, adipogenesis, obesity

## Abstract

Selenium (Se) homeostasis is tightly related to carbohydrate and lipid metabolism, but its possible roles in obesity development and in adipocyte metabolism are unclear. The objective of the present study is to review the current data on Se status in obesity and to discuss the interference between Se and selenoprotein metabolism in adipocyte physiology and obesity pathogenesis. The overview and meta-analysis of the studies on blood Se and selenoprotein P (SELENOP) levels, as well as glutathione peroxidase (GPX) activity in obese subjects, have yielded heterogenous and even conflicting results. Laboratory studies demonstrate that Se may modulate preadipocyte proliferation and adipogenic differentiation, and also interfere with insulin signaling, and regulate lipolysis. Knockout models have demonstrated that the selenoprotein machinery, including endoplasmic reticulum-resident selenoproteins together with GPXs and thioredoxin reductases (TXNRDs), are tightly related to adipocyte development and functioning. In conclusion, Se and selenoproteins appear to play an essential role in adipose tissue physiology, although human data are inconsistent. Taken together, these findings do not support the utility of Se supplementation to prevent or alleviate obesity in humans. Further human and laboratory studies are required to elucidate associations between Se metabolism and obesity.

## 1. Introduction

Selenium (Se) is a metalloid discovered by the Swedish chemist Jöns Jakob Berzelius in 1817 and recognized as an essential trace element in higher animals in 1957 [1]. Physiological functions of Se are mainly mediated through Se-containing proteins, selenoproteins, containing Se in the form of selenocysteine [2], although inorganic Se may also possess direct biological activity [3]. Selenoproteins, such as glutathione peroxidases (GPXs) and thioredoxin reductases (TXNRDs), were initially recognized as antioxidants. Later, it was found that selenoproteins intricately regulate the functioning of the endocrine system and intracellular signaling [4].

Today, it is recognized that adequate Se intake is essential for immune [5], endocrine [6], cardiovascular [7], reproductive [8], and nervous systems [9]. Severe Se deficiency may cause Kashin-Beck and Keshan diseases [10]. Keshan disease is endemic cardiomyopathy associated with selenium deficiency and is characterized by a spectrum of cardiovascular disorders of varied intensity from cardiogenic shock, hypotension and arrhythmia in acute type to fatigue and asymptomatic course in chronic and latent types, respectively [11]. In turn, Kashin-Beck disease is an osteoarthropathy characterized by alteration of the epiphyseal plate and articular surface resulting in permanent deformation [12]. Less pronounced Se insufficiency may be associated with increased risks of cancer, and cardiovascular diseases [10,13]. Dietary selenium intake is considered as the key determinant of selenium status. Specifically, meat and other animal products (liver, kidneys), cereals, *Allium* (onion, garlic) and *Brassica* (broccoli, cauliflower) species, and yeast contribute significantly to daily selenium intake, with the highest Se content is found in Brazil nuts [14]. However, certain internal factors may also affect dietary Se intake by limiting its bioavailability. Particularly, gut microbiota may mediate the effects of dietary Se on Se status of the host through modulation of selenoprotein metabolism [15]. Therefore, alteration in gut microbiota in a number of intestinal (inflammatory bowel disease, Crohn’s disease, colon cancer) and metabolic (e.g., obesity) diseases may also modify dietary Se bioavailability and status.

At higher doses, Se may be toxic, and the range of optimal intakes is rather narrow, showing some evidence of a U-shaped association with human health [16]. Specifically, chronic low-dose Se overexposure may potentiate diabetes [17,18], along with cardiovascular diseases [19] and neurodegeneration [20].

Se plays a significant and complex role in modulating insulin signaling, and consequently carbohydrate and lipid metabolism [21]. Although physiological levels of Se may exert an insulin-mimetic effect, supraphysiological high doses of Se are reported to impair insulin synthesis and induce insulin resistance in different species [22,23,24,25,26]. Se overexposure was shown to increase type 2 diabetes mellitus (DM2) risk at a wide range of concentrations, also encompassing levels of intake so far considered to be safe [16,18]. Despite the presence of a plethora of studies of the role of Se in diabetes, its involvement in obesity, is insufficiently studied. Data from observational studies regarding the association between Se and obesity [27] and metabolic syndrome (MetS) [28] are rather contradictory.

Moreover, recent findings demonstrating the high rate of selenoprotein expression in adipose tissue in both healthy [29] and obese conditions [30] are also indicative of the significant roles of Se in adipocyte biology. However, the existing data on the involvement of particular selenoproteins in adipose tissue physiology and pathology appear insufficient. Therefore, the objective of the present study was to review the current data on Se status in obesity, as well as to discuss the relationship between Se and selenoprotein metabolism with adipocyte physiology and its possible disturbances in obesity pathogenesis. Where possible, we carried out a meta-analysis of mean differences of Se exposure according to different indicators (biomarker levels, GPX activity, and selenoprotein P (SELENOP) levels) and outcome, using a methodology specified in detail elsewhere [31]. In particular, we compared Se exposure in normal-weight versus over-weight individuals, subjects with and without MetS/obesity. Since units of measurement may differ across the selected studies, we computed the standardized mean differences (SMD) and corresponding 95% confidence intervals (CIs), using a random-effect model to account for heterogeneity (*I*^2^) in study-specific results.

## 2. A Brief Introduction into Selenoprotein Function

Detailed insight into the biochemistry and molecular biology of selenoproteins has been provided in a number of excellent reviews [1,2,4,32,33,34]. Therefore, only a brief introduction into general functions of selenoproteins will be provided herein.

Selenoproteins represent a wide heterogenous group of proteins containing selenium in the form of selenocysteine (Sec). More than 50 selenoprotein families have been identified to date, although only 25 of them are present in humans [32]. However, the specific reactions have been estimated only for GPXs, TXNRDs, MSRB1, DIOs, and SPS2 [4]. Glutathione peroxidase 1 (GPX1) was the first selenoprotein identified with a demonstrated function in humans. Generally, GPX-specific reaction is reduction of H2O2 and other hydroperoxide using reduced glutathione (GSH) as thiol donor [35]. Therefore, physiological role of GPXs is mediated by its involvement in redox regulation of cellular functions [36]. In human, GPX1, GPX2, GPX3, GPX4, and GPX6 are the Se-containing GPX isoforms and characterized by different localizations in cytoplasm, intestine, plasma, cytoplasm/mitochondria/nucleus and embryos/adult olfactory epithelium, respectively, whereas GPX4 plays a role of phospholipid hydroperoxide. Thioredoxin reductases (TXNRDs) are selenoproteins playing the next stage in antioxidant defense by maintaining the redox state of proteins by NADPH-dependent reduction of thioredoxin [37]. The three isoforms, TXNRD1, TXNRD2, and TXNRD3, are found in the cytoplasm and nucleus, mitochondria, and testes [33]. Similarly to TXNRDs, selenoprotein MSRB1 is also involved in the maintenance of redox state of the proteins through reduction of methionine sulfoxides (-Met-SO) to methionine (-Met) [38]. Deiodinases (DIOs) play an essential role in the metabolism of thyroid hormone and its metabolites through 5′-deiodination in thyroid, as well as multiple peripheral tissues [39]. In turn, selenophosphate synthetase 2 (SPS2) catalyzes the synthesis of selenophosphate from selenide, playing an critical role in selenoprotein biosynthesis [40].

Selenoprotein P (SELENOP), being the most abundant selenium-containing glycoprotein containing ten selenocysteine residues, is considered to be a Se transporter which also possesses direct antioxidant activity [41,42]. Endoplasmic reticulum (ER)-resident selenoproteins represent a significant subgroup of selenoproteins including selenoprotein F (SELENOF), selenoprotein K (SELENOK), selenoprotein M (SELENOM), selenoprotein N (SELENON), selenoprotein S (SELENOS), and selenoprotein T (SELENOT), and are involved to a different extent in redox sensing and regulation, protein folding, and calcium homeostasis [33]. Impairment of these selenoprotein-dependent functions in ER plays a significant role in the formation of ER stress [33] known to be associated with misfolded protein accumulation in ER and downstream signaling [43]. The latter was shown to be involved in pathogenesis of multiple diseases including diabetes, neurodegeneration, cancer, and viral infections [43].

The function of several selenoproteins is still not appreciated. SELENOH, SELENOT, SELENOV and SELENOW were shown to be related to a thioredoxin-like family of selenoproteins, thus being involved in redox-regulation and sensing [44]. Selenoprotein O (SELENOO) was identified as a mitochondrial CxxU-containing redox-active protein expressed in multiple tissues [45]. Selenoprotein I (SELENOI) also known as ethanolamine phosphotransferase 1 (EPT1) was shown to be essential in myelination hypothetically through its role in phospholipid biosynthesis [46].

Therefore, the existing data demonstrate that selenoproteins are both directly and indirectly involved in a myriad of metabolic pathways. However, the particular patterns of altered selenoprotein expression and function and their roles in pathogenesis of various diseases are yet to be estimated [47].

## 3. Se Status in Obesity

Se status in obesity has been investigated in a number of studies using various biomarkers including more routine ones like Se levels in different tissues, as well as functional biomarkers, such as GPX activity and SELENOP levels [48] (Tables S1–S3).

### 3.1. Se Blood Levels and Intake Patterns

In a study by Zhong et al. (2018) involving 6440 men and 6849 women, serum Se levels were inversely associated with increased body mass index (BMI) with −4 ng/mL (95% CI: −5.5 to −1.6 ng/mL) and difference between the highest and the lowest quartiles was statistically significant. Moreover, high (Q4) percent body fat (BF%) was related to a decrease in serum Se levels by −1.7 ng/mL (95% CI: −4.2 to 0.7 ng/mL) (nonsignificant) and −4.5 ng/mL (95% CI: −7.0 to −1.9 ng/mL) in men and women, respectively [49]. Se was found to be significantly reduced, by ~15%, in women with morbid obesity when compared with the lean controls [50]. A detailed examination of 573 Madrid schoolchildren demonstrated that in overweight children (BMI > P_85_) both serum Se and Se intake were 14% and 27% lower as compared with normal-weight children. Moreover, increased BMI was considered as a significant predictor of blood Se deficiency (odds ratio (OR) = 1.5031; 95% CI: 1.3828 to 1.6338) and low Se intake (OR = 0.9862; 95% CI: 0.9775 to 0.9949) in logistic regression models [51]. These findings generally corroborate the observation by Błażewicz et al. (2015), who demonstrated reduced serum and urinary Se levels in obese children [52].

**Figure 1:**
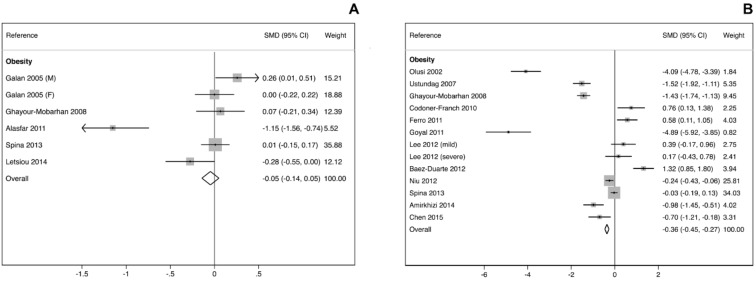
Mean difference in individual studies and summary standardized mean difference (SMD) of selenium (Se) levels (**A**) and glutathione peroxidase (GPX) activity (**B**) in patients with obesity compared with controls, adult population.

In meta-analysis comparing mean levels of Se in blood/serum, we found a negative mean difference for obese compared to healthy adult subjects. Conversely, we found that Se levels were higher in subjects with MetS (Figure 1A). We also revealed similar associations between Se status and obesity in children (Supplemental Figure S1).

At the same time, Se deficiency (defined as serum Se < 0.75 μmol/L) was 2% and 11–15% before and after bariatric surgery in morbidly obese patients [53]. Correspondingly, examination of obese patients considering bariatric surgery revealed a rather low prevalence of Se deficiency in women (3.6%) and men (1.8%) [54]. In an adult French population serum Se levels were not associated with BMI, but were associated with serum cholesterol levels [55]. Accordingly, BMI was not considered to be a significant predictor of Se distribution [56].

In contrast, increased Se status was observed in obese individuals. For example, hair Se levels were higher in subjects with obesity, although no effect of diabetes was observed in this research [57]. In a recently published cross-sectional study in an adult Chinese population, serum Se concentration was positively associated with the risks of both hyperglycemia and dyslipidemia, and both these metabolic disorders had higher BMI than their respective controls [58]. Although high serum Se levels were associated with increased BMI (23.9 ± 2.9 (Q1) vs. 25.5 ± 4.0 (Q4), *p* < 0.001), as well as other markers of MetS, the association between increased Se supply and insulin resistance was found to be obesity-independent [59]. Elevated plasma Se was found to be associated with increased BF% and BMI in patients with DM2 [60].

In a general Japanese population plasma Se levels were directly associated with waist circumference (r = 0.329; *p* = 0.029) but not BMI (r = 0.225; *p* = 0.187), whereas circulating SELENOP levels were unrelated to anthropometric parameters [61].

In the IMMIDET study MetS was shown to be directly associated with increased plasma Se levels (OR = 1.33 (1.06–1.67)) after adjustment for multiple confounders [62]. This was confirmed in another study, showing a stronger association between elevated serum Se and MetS in women [63]. A study by Mutakin et al. (2013) aimed at assessment of the impact of MetS components in obesity found no substantial difference in both Se levels and GPX activity between obesity, obesity with one component of MetS, as well as verified MetS. In patients with visceral obesity plasma Se levels significantly correlated with high density lipoprotein cholesterol (HDL-C) and fatty acid binding protein 4 (FABP4), whereas in MetS plasma Se levels were inversely associated with circulating macrophage chemoattractant protein 1 (MCP-1). GPX activity directly correlated with HDL-C in obesity plus one component of MetS and were inversely associated with FABP4 in obese patients [64].

Certain studies also demonstrated the association between dietary Se and obesity. Specifically, higher Se intake both in men and women was associated with lower BMI, waist circumference, and body fat, being responsible for up to 27% of body fat percentage variability [65]. Correspondingly, dietary Se intake in young healthy women was inversely associated with retinol binding protein 4 (RBP4) known to be a metabolic risk marker [66].

In addition, in Se-sufficient obese women Brazil nut intake resulted in a significant increase in plasma and red blood cell (RBC) or RBC Se content, SELENOP levels, and GPX1 activity, as well as proinflammatory cytokine levels. Particularly, the change in plasma and RBC Se, as well as SELENOP level, was directly associated with tumor necrosis factor (TNF) α, interleukin (IL) 6, IL10, Toll-like receptor (TLR) 2, and TLR4 gene expressions [67].

Therefore, the existing studies on blood selenium levels in obesity provide highly heterogenous results not only regarding the patterns of Se status in overweight and obese subjects, but also its association with metabolic risk markers.

### 3.2. GPX Activity and Levels

In the study by Karaouzene et al. (2011), GPX activity significantly decreased in obese patients irrespective of age, whereas superoxide dismutase (SOD) and catalase levels were differentially associated with obesity in young and old obese subjects [68]. Patients with morbid obesity (BMI > 40 kg/m^2^) were characterized by a 14% decrease in GPX activity, which was inversely associated with BMI values [69]. In addition to the inverse association between BMI and erythrocyte GPX activity, Amirkhizi et al. (2014) found a negative impact of abdominal obesity on GPX activity as compared to normal type of fat distribution [70]. It is also noteworthy that in obese patients from the United Kingdom, reduced serum GPX activity was associated with elevated Se levels, whereas these differences were less pronounced in the presence of other components of MetS [71]. Despite the absence of group differences, plasma GPX activity was directly interrelated with waist-to-hip ratio values in obese patients with sleep apnea [72]. While only GPX activity was shown to be inversely associated with obesity (BMI > 30 kg/m^2^), increased waist circumference and waist-to-hip ratio was found to be negatively correlated with GPX activity and to a lesser extent RBC Se levels, whereas no significant dependence was observed for plasma Se concentrations [73].

RBC GPX activity was 26% higher in obese Brazilian women as compared to the controls [74]. RBC GPX activity was characterized by a considerable increase in obese and centrally obese diabetic subjects in parallel with oxidative stress markers [75]. Examination of overweight and obese subjects from Central Mexican population also demonstrated a significant 22% increase in serum GPX3 levels, being also associated with insulin sensitivity and atherogenicity index [76]. These findings corroborate earlier data of increased plasma GPX activity in severely obese and insulin resistant (homeostatic model assessment for insulin resistance index (HOMA-IR) > median) patients [77].

In contrast to the findings on Se levels, mean blood GPX activity were generally decreased in obese individuals (Figure 1B).

Data on blood GPX activity in children are also contradictory. Particularly, the existing reports demonstrate a significant decrease [78,79] and increase [80,81]. It is also interesting that erythrocyte GPX activity was found to be increased only in overweight children, whereas in obese examinees it was nearly analogous to the control values [82]. Similarly, plasma GPX levels in obese insulin resistant children were found to be decreased as compared to lean and obese children without insulin resistance by 50% and 31%, respectively [83]. The standardized mean difference demonstrated small increase in obese children, although included studies demonstrated highly heterogenic results (Supplemental Figure S1B).

GPX levels and activity were also increased following weight reduction and weight loss therapy. Particularly, serum GPX activity was found to be increased in response to weight loss therapy, being inversely associated with IL6 levels [84]. Both dietary intervention and/or orlistat treatment in obese women resulted in a significant increase in blood GPX activity [85]. Correspondingly, 24 weeks of insulin treatment in obese and non-obese diabetics resulted in a significant increase in GPX activity as compared to the baseline [86]. Moreover, improvement of GPX activity was tightly associated with metabolic parameters and pathogenetic mechanisms in obesity. Particularly, weight loss therapy resulted in a significant increase in lymphocyte GPX1 activity, being associated with reduced proinflammatory cytokine and adipokine synthesis, endoplasmic reticulum (ER) stress (ERS), lower mitochondrial reactive oxygen species (ROS) production, and improvement of membrane potential [87]. A 12-week histidine treatment was shown to improve anthropometric and metabolic parameters in obese women with MetS, as well as to increase GPX activity by 10% [88]. These findings correspond to the earlier observed positive association between GPX activity and serum histidine levels [89].

Multiple studies have demonstrated that GPX polymorphisms may be associated with obesity. Particularly, GPX1 Pro198Leu polymorphism (rs1050450) was found to be associated with increased body fat mass (CC/TT) in Japanese women [90]. GPX1 Pro200Leu polymorphism (rs1050450) was also shown to be associated with morbid obesity, but not (pre)diabetes in obesity in a Mexican population [91]. In addition, polymorphisms rs757228 and rs8103188 of GPX4 (negatively), as well as variants rs445870 of GPX5 and rs406113 of GPX6 (positively), were associated with obesity in prepubertal Spanish children [92]. In addition, it has been also demonstrated that GPX1 Pro198Leu variants may affect the efficiency of Brazil nut supplementation in obese women [93].

### 3.3. Circulating SELENOP Levels

**Figure 2:**
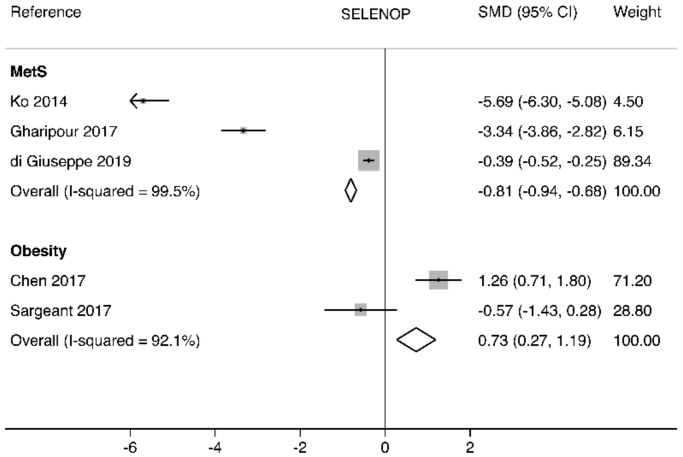
Mean difference in individual studies and summary standardized mean difference (SMD) of Selenoprotein P (SELENOP) levels in patients with obesity, or metabolic syndrome (MetS) compared with controls, adult population.

SELENOP is considered as one of the most sensitive functional markers of Se status, being a transport form of Se in human blood [94]. High SELENOP levels (11.72 mg/mL Q4 vs. 2.86 mg/mL Q1) were associated with 11%, 35%, and 28% lower BMI, visceral adipose tissue (VAT), and subcutaneous adipose tissue (SAT) levels, respectively, being also associated with lower prevalence of MetS and nonalcoholic fatty liver disease (NAFLD) [95]. Although Se levels were found to be higher in patients with MetS, SELENOP level was associated with reduced MetS risk (0.995; 95%CI = 0.989–1.00, *p* < 0.04), being also inversely associated with waist circumference (OR = 0.995 (95% CI = 0.990–1.00) [96]. In Korean children MetS was associated with 42% lower circulating SELENOP levels. The latter were also significantly negatively associated with anthropometric markers of obesity, blood pressure, triglycerides, as well as insulin resistance, being positively correlated with HDL-C. Moreover, the highest tertile of serum SELENOP levels was associated with reduced risk of MetS in non-adjusted (OR = 0.18 (95%CI: 0.09–0.37)) and adjusted (OR = 0.05 (95%CI: 0.00–0.96)) models [97]. Moreover, in patients who underwent gastric bypass surgery, SELENOP levels significantly increased in 1 and 9 months after surgery, being characterized by a significant inverse association with serum (GGT) activity, whereas the amplitude of SELENOP changes negatively correlated with ΔHOMA-IR [98]. Moreover, dietary pattern characterized by high intake of fruit, vegetables, and antioxidant beverages, reduced both VAT and SAT mass, as well as the risks of MetS and DM, being also associated with elevation of SELENOP levels [99]. The standardized mean difference showed opposite results for SELENOP levels, with negative SMD for higher BMI, while a positive one for obesity (Figure 2).

In contrast, examination of Korean non-diabetic adults with visceral obesity and NAFLD demonstrated that increased SELENOP levels were associated with visceral obesity, as well as NAFLD, increasing the risk of the latter in regression models [100]. Moreover, circulating SELENOP levels correlated with HOMA-IR and were more than 3-fold higher in patients with obesity (52.3 ± 39.1 vs. 14.5 ± 12.8 μg/mL, *p* < 0.001), whereas SAT SELENOP mRNA expression was significantly associated with BMI [101]. At the same time, Sargeant et al. (2017) demonstrated that overweight/obese subjects did not differ from the normal-weight ones in both basal and post-exercise (60 min of moderate-intensity treadmill exercise) SELENOP levels [102].

## 4. Se in Adipogenesis and Adipocyte Signaling Pathways

Adipogenesis is the most vulnerable period of adipose tissue physiology, and its impairment plays a significant role in pathogenesis of obesity [103]. Recent studies have demonstrated that Se may be a potential agent having a significant impact on adipogenesis and its regulation through signaling pathways [34]. However, the existing data demonstrate differential influence of Se doses (and possibly species applied) on adipogenesis.

Particularly, Se treatment in nearly physiological range (0, 5, 10, 20 ng/mL) significantly up-regulated 3T3-L1 preadipocytes differentiation by 20% through modulation of cell cycle signaling (G2/M progression) by increasing cyclin-dependent kinase (CDK1 and CDK2) expressions through down-regulation of CDK inhibitors (CDKNs) p21 and p27. In addition, phosphatidylinositol-3-kinase (PI3K)/protein kinase B (Akt or PKB) but not mitogen-activated protein kinase (MAPK) 1 (ERK) pathway activation was observed. Se-induced increase in preadipocyte migration was shown to be associated with up-regulation of matrix metallopeptidase (MMP) 2 and MMP9 [104]. At the same time, another study demonstrated that Se exposure in the physiological range (0-50-150 µg/L) in adipocytes stimulated an exit from the cell cycle and further adipogenic differentiation through modulation of cell cycle control genes including *CDKN1*, *CDKN2B*, fibroblast growth factor 2 (*FGF2*) (all up-regulation), as well as cyclin E2, B3, and D2 (all down-regulation). Notably, these changes occurred in parallel with up-regulation of TXNRD, methionine sulfoxide reductase 1 (MSRB1), selenoprotein O (SELENOO), and lipid biosynthesis genes. These effects were observed in chicken embryonic fibroblasts from 6-day-old but not 9–12-day-old embryos, being indicative of Se efficiency only at earlier stages of adipogenesis [105]. In addition, PI3K/Akt activation induced by treatment with physiological dose of sodium selenite (2 µM) was associated with up-regulation of anti-apoptotic Bcl2, restoration of mitochondrial membrane potential, and increasing glucose uptake in HT1080 cells [106].

**Figure 3:**
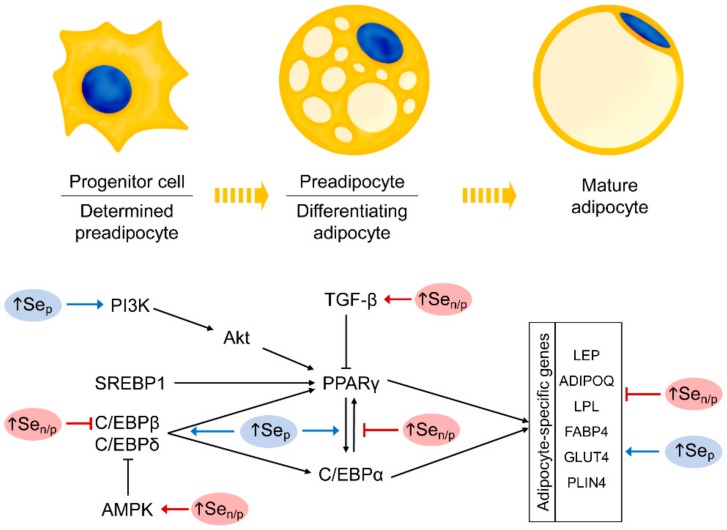
Differential effects of physiological (blue, Se_p_) and supraphysiological (red, Se_n/p_) doses of selenium on adipocyte differentiation mechanisms in vitro. Briefly, selenium in the physiological range possesses overall adipogenic effect by activation of PPAR and C/EBPα signaling through positive modulation of PI3K/Akt and C/EBPβ/δ pathways, resulting in up-regulation of adipocyte-specific genes. Oppositely, Se overload exerts antiadipogenic activity through suppression of PPAR and C/EBPα expression though several mechanisms including reduction of C/EBPβ expression as well as activation of AMPK and TGF-β. ADIPOQ, adiponectin; Akt, protein kinase B; AMPK, 5′AMP-activated protein kinase; C/EBP, CCAAT-enhancer binding protein; FABP4, fatty acid binding protein 4; GLUT4, glucose transporter type 4; LEP, leptin; LPL, lipoprotein lipase; PI3K, phosphatidylinositol-3-kinase; PLIN4, perilipin 4; PPAR, peroxisome proliferator-activated receptor; SREBP1, sterol regulatory element-binding protein 1; TGF-β, transforming growth factor β.

Differential effects were observed upon selenate (SeO_4_^2−^) exposure (Figure 3). Particularly, Se as sodium selenate at supraphysiological doses (100, 200, 400 µM) was shown to down-regulate adipocyte differentiation and induce apoptosis in 3T3-L1 cells through 5′AMP-activated protein kinase (AMPK) activation and phosphorylation of its substrate acetyl-CoA carboxylase (ACC) [107]. Another study demonstrated that antiadipogenic effect of supraphysiological but non-cytotoxic doses of NaSeO_4_ (1, 10, 25, 50 µM) might be associated with reduced peroxisome proliferator-activated receptor (PPAR)γ and CCAAT-enhancer binding protein (C/EBP)β expressions without any cytotoxic effects [108]. Correspondingly, NaSeO_4_ exposure in the same dose (50 µM) inhibited adipocyte differentiation suppressing PPARγ, C/EBPα, and leptin gene expression. The authors proposed that antiadipogenic effect of Se might be associated with up-regulation of selenoprotein S (SELENOS) and attenuation of ERS, especially at early stages of adipogenesis [109]. It is notable that selenate but not selenite or methylseleninic acid treatment in supraphysiological doses (5, 10, 25, 50 µM) was shown to inhibit adipogenic differentiation of preadipocytes through activation of transforming growth factor-β1 (TGF-β1) and interference with C/EBP signaling without a reduction in cell viability [110]. However, antiadipogenic effect of selenate (50 µM) was shown to be limited to early stages (0–2 days) of adipogenesis in 3T3-L1 cells. This was consistent with a decrease in adipogenesis without cytotoxicity induced by supranational doses of selenate [111].

Treatment with physiological doses of selenocysteine (50 and 100 nM) of adipose tissue-derived mesenchymal stem cells significantly increased cell survival and down-regulated prooxidant and proinflammatory pathways, thus possessing a protective effect on replicative senescence of adipose tissue-derived mesenchymal stem cells. These effects were accompanied by differential selenoprotein response characterized by up-regulation of GPX1 and reduced SELENOO and MSRB1 expressions [112].

Results from animal studies also demonstrate a significant impact of Se treatment on diet-induced obesity in laboratory animals. Particularly, sodium selenate in the supraphysiological dose of 0.72 mg/kg/day (8 weeks) significantly reduced adiposity in HFD-fed mice through down-regulation of PPARγ signaling and a concomitant reduction in diacylglycerol *O*-acyltransferase (DGAT) 2 and leptin expression, as well as improvement of insulin sensitivity [113]. In turn, physiological intake of sodium selenite (0.3 mg/kg diet for 4 weeks) in HFD-fed rats potentiated the effects of probiotic treatment on down-regulation of fatty acid synthase (FAS), lipoprotein lipase (LPL), PPARγ, and sterol regulatory element-binding protein (SREBP) 1, whereas expression of genes involved in lipid catabolism was increased in response to treatment [114]. Se-enriched Enterobacter exopolysaccharides significantly reduced adipose tissue inflammation by down-regulating IL6 and TNF expression both in vivo (HFD-fed diabetic KKAy mice) and in vitro (3T3-L1 adipocytes) through an AMPK/SirT1 pathway [115]. Moreover, sodium selenite injected intraperitoneally at a dose of 5 µM/kg b.w. for 2 weeks significantly enhanced fatty acid β-oxidation through up-regulation of β-oxidation gene expression including acyl-CoA dehydrogenase thus preventing adipocyte hypertrophy and fatty liver development in the OLETF rat model [116]. It is notable that the period of Se exposure may have a significant effect on adipogenesis regulation. Particularly, in high fat diet (HFD)-fed mice pretreatment with supraphysiological Se levels (29.13 mg/L in drinking water for 2–4 months) significantly increased adipocyte differentiation and reduced ectopic lipid accumulation involving peroxisome proliferator-activated receptor (PPAR) γ and CCAAT-enhancer binding protein (C/EBP) α/β modulation, whereas Se posttreatment up-regulated lipolysis and subsequent ectopic lipid accumulation through protein kinase A (PKA) / hormone-sensitive lipase (HSL) pathway. Both effects were found to be associated with up-regulation of SELENOP and GPX1 expressions [117].

Generally, the reviewed data on the effects of Se species (selenite and selenate) exposure on adipogenesis seems contradictory especially with respect to in vitro studies. Such inconsistency may hypothetically be explained by the difference in applied Se doses. Particularly, sodium selenite [85,86,87] was mainly used in physiological and nearly physiological doses [118] resulting in up-regulation of adipogenesis, whereas treatment with sodium selenate [107,108,109,110,111] accounted for supranutritional exposure. These findings may be indicative of the differential role of physiological and supraphysiological Se levels in regulation of adipogenesis. Hypothetically, in the physiological range Se may be considered as essential factor contributing to increased adipogenesis, whereas at higher doses it prevents adipocyte differentiation and hypertrophy (Figure 3). It is important to mention that antiadipogenic effect of high selenium exposure (~50 µM) is unlikely to be associated with cytotoxicity, as no significant alteration of cell viability was observed at this dose [108,110,111].

Both in vivo and in vitro studies demonstrate that the impact of both selenate and selenite on adipogenesis may be differentially mediated through PPARγ modulation, being a key regulator of adipogenic differentiation [119]. It is hypothesized that Se may have anti-obesity effects through modulation of PPARγ signaling and development of lipophilic Se compounds capable of binding PPARγ is of particular interest [120].

Earlier studies demonstrated that Se (1 mM) may possess insulin-mimetic effect in adipocytes [121]. Specifically, sodium selenate treatment increased phosphorylation of insulin receptor β-subunit, insulin receptor substrate 1 (IRS1), and MAPK in 3T3-L1 and primary rat hepatocytes in a wide range of concentrations, as well as increased 2-deoxy-D-glucose uptake by 3T3-L1 adipocytes (0.1, 0.25, 0.5, and 1.0 mM) [122]. Se was also shown to possesses both insulin-like and non-insulin-like action in 3T3-L1 adipocytes through stimulation of glucose transport and reduction of lipolysis due to up-regulation of PI3K, Akt phosphorylation, and glucose transporter 1 (Glut1) translocation [123]. It has been also demonstrated that sodium selenate (1 mM, 0–30 min) is capable of MAPK and S6 kinase activation in adipocytes, providing a longer effect as compared to insulin [124].

Certain studies demonstrated that Se has a significant impact on lipolysis, a process counteracting adipose tissue hypertrophy. Specifically, in an early study 1 mM sodium selenate treatment increased LPL activity in isolated rat adipose tissue through a Ca^2+^ and inositol 1, 4, 5-trisphosphate (IP3)-dependent process [125]. Although high-Se treatment (0.50 mg/kg, 16 weeks) did not alter selenoprotein (GPX and TXNRD) expression and activity in VAT, Se exposure resulted in a 60% increase in SREBP expression and a tendency to LPL up-regulation due to impaired AMPK activity [126]. In addition, high Se (10 µM as SeO_3_^2−^) exposure was also shown to deplete chromium and impair redox homeostasis by upregulation of GPX and SELENOP ultimately leading to increased lipolysis and hepatic insulin resistance [127].

Altogether, these findings demonstrate that Se may not only modulate proliferation of preadipocytes, but also determine their adipogenic differentiation with lipid accumulation, interfere with insulin signaling, and regulate lipolysis and other cell-specific functions. However, the observed effects were characteristic for supraphysiological Se exposure for both cells (>50 nM) and animals (>0.2 mg/kg diet). At the same time, whether these effects persist within the physiological range of exposure seems underestimated.

## 5. Adipose Tissue as a Target for Se Activity: Adipocyte Selenoproteins

Selenium levels in adipose tissue were found to be much lower as compared to kidney, liver, heart, and muscle [128]. Specifically, Se content in lean adipose tissue (<0.2 µg/g) was found to be more than 4-fold lower as compared to liver (~0.8 µg/g) [129]. At the same time, adipose tissue content was shown to respond to modulation of dietary Se intake. In particular, administration of diphenyl diselenide dissolved in canola oil resulted in a significant increase in adipose tissue Se content both in rats and mice in 1 and 5 days after exposure followed by a reduction to the baseline levels [130]. Adipose tissue selenoproteome was also shown to respond readily to modulation of Se status. Particularly, maintenance of chickens on low-Se diet results in a significant time-dependent decrease in expression of all 25 tested selenoproteins including GPXs, TXNRDs, DIOs, SELENOP, etc. [131].

These findings are indicative of active selenium transport into adipose tissue, although the particular mechanisms remain unclear. SELENOP-mediated Se transport is known to be one of three ways of Se transport also including Se uptake via anion transporters (inorganic Se) and methionine transporters (selenomethionine) [132]. SELENOP uptake was shown to occur through receptor mediated endocytosis via LRP2 (megalin) and LRP8 (ApoER2) [132]. Correspondingly, SELENOP transports Se to peripheral organs expressing LRP2 or LRP8 [133]. Earlier studies revealed significant adipocyte LRP2 expression [134]. Although data from adipocytes are unavailable, it is notable that PPARα/γ up-regulates LRP2 expression in epithelium [135]. This observation may provide a link between adipogenesis accompanied by PPARγ activation and increased selenoprotein uptake and synthesis. Such a hypothesis is indirectly supported by the observation of megalin expression in preadipocytes [136], but not mature adipocytes [137]. LRP8 expression was shown to be overexpressed during human adipocyte maturation [138]. It was also found to be higher in extra-hepatic adipose tissue as compared to liver in ducks [139]. However, no LRP8 expression was observed in adipose tissue of mice [140]. The human gene database GeneCards provides data on human adipocyte LRP2 and LRP8 expression. Specifically, the *Lrp2* gene (GCID:GC02M169127) was found to be expressed in human adipocytes, although at a much lower rate as compared to certain other tissues (e.g., kidney, brain, thyroid). At the same time, *Lrp8* gene (GCID:GC01M053243) was found to be overexpressed in human adipocytes followed by bone marrow mesenchymal stem cells and heart. Therefore, the existing limited body of data allow to propose the leading role of LRP8 in Se uptake by adipocytes, although further studies are required to support this hypothesis.

Despite a relatively low Se content in adipose tissue [128,129] mapping of murine adipocyte proteome demonstrated high abundances of selenoproteins in gonadal white adipose tissue (WAT) [29]. Importance of Se for adipose tissue biology was also supported by the observation of the role of Se-binding protein 1 (SELENBP1) as a marker of mature adipocytes, being associated with lipid accumulation and other functional markers of adipocyte differentiation [141]. Peinado et al. proposed that modulation of SELENBP1 expression in VAT may be associated with HIF-1 signaling in obesity [142]. Furthermore, adipogenic differentiation of human bone marrow stromal stem cells was shown to be associated with up-regulation of SELENOF, -M, -P, and SELENBP1, along with FABP4, ADIPOQ, and C/EBPα. Se metabolism pathway was considered as one of the top ten enriched pathway categories [143]. Correspondingly, in pigs feeding a HFD reduced selenoprotein mRNA expression in perirenal adipose tissue (SELENOH, -K, -P, -V, and -W) and SAT (SELENOI, -M, and MSRB1) [30]. Although multiple selenoproteins have been identified in adipose tissue, their particular roles in regulation of adipocyte metabolism and obesity pathogenesis are still unclear.

### 5.1. Selenoprotein P (SELENOP)

SELENOP gene was shown to be expressed in all rat adipose tissue depots, being indicative of its significant role in adipocyte physiology [144]. Reduced SELENOP levels were found to be associated with impaired adipogenesis due to antioxidant selenoprotein insufficiency (GPX), oxidative stress, and preadipocyte inflammation. It is also notable that cocultivation of *Selenop*-knockout (KO) (*Selenop*^−/−^) cells with intact 3T3-L1 cells resulted in inhibition of adipocyte differentiation [145]. Moreover, Selenop deficiency was shown to prevent diet-induced increase in adipose tissue accumulation and adipocyte hypertrophy, although caloric intake in *Selenop*^−/−^ mice exceeded the wildtype values [146]. These findings demonstrate that Selenop is an essential factor of adipogenesis. This suggestion is confirmed by the observation of *Selenop* gene up-regulation in C/EBPα-overexpressing NIH3T3 cells characterized by induction of adipogenic differentiation [147]. It is also notable that antiadipogenic effect of selenate was dependent on *Selenos* and *Selenop* gene expression, whereas attenuation of antiadipogenic effect at postmitotic growth arrest (days 2–4) was also associated with selenoprotein gene downregulation [148].

Adipocyte SELENOP also significantly responded to proinflammatory stimuli involved in pathogenesis of obesity and associated metabolic disturbances. Particularly, differentiated adipocytes responded to omentin exposure in vivo by a significant decrease in SELENOP expression in parallel with proinflammatory response [149]. Correspondingly, *Selenop* gene expression in 3T3-L1 adipocytes was reduced in response to TNFα or H_2_O_2_ treatment, being indicative of the link between adipose tissue inflammation and oxidative stress in obesity and altered selenoprotein metabolism [150]. Hypoxia-induced increase in proinflammatory cytokine (IL6 and MCP1) expression in proliferating preadipocytes was also associated with negative regulation of Selenop levels [151].

The association of obesity with alteration of *Selenop* expression in adipose tissue also supported the essential functions of Selenop. Particularly, a significant reduction in *Selenop* gene expression in adipose tissue of obese (ob/ob), HFD-fed, and Zucker rats, as well as insulin-resistant patients, was demonstrated [150]. Leptin treatment in ob/ob mice resulted in the overall shift to lipid catabolism genes involving inhibition of SREBP1 downstream signaling, as well as up-regulation, of Selenop and SREBP1 expression in the liver [152]. In contrast, *Selenop* expression was found to be 2-fold higher in obese adipose tissue of OLETF rats [144].

### 5.2. Selenoprotein S (SELENOS)

Given a role of SELENOS as an ER-resident antioxidant it is highly likely that its role in adipogenesis will be mediated through modulation of ERS known to be involved in regulation of adipocyte physiology and pathology [153]. Particularly, it has been demonstrated that SELENOS reduces ERS and ERS-dependent adipogenesis resulting in decreased C/EBPα levels and other adipocyte secretion products, whereas dexamethasone-induced proteosomal SELENOS degradation at early phase of cell differentiation promotes adipogenesis [154]. At the same time, Selenos knockdown was shown to result in increased apoptotic cell death through IRE1α-sXBP1-p-JNK pathway and ERS [155]. Modulation of SELENOS-induced ERS may also underlie selenate-induced inhibition of adipogenesis in 3T3-L1 preadipocytes without cytotoxicity [156].

SELENOS was shown to be mutually interrelated with PPARγ signaling. Particularly, an inverse association between SELENOS expression and PPARγ activation during adipogenesis was demonstrated [157]. In turn, PPARγ-mediated SELENOS and SELENOK ubiquitination and degradation through direct interaction with selenoproteins via ligand-binding domain is essential for adipocyte differentiation [158].

SELENOS expression in adipose tissue was found to be slightly but significantly increased in obese patients, being also significantly correlated with anthropometric measures of obesity and insulin resistance. In vitro study using isolated human adipocytes demonstrated that insulin up-regulates SELENOS expression, thus providing a link between insulin resistance and SELENOS expression in obesity [159].

### 5.3. Glutathione Peroxidases (GPXs)

Analysis of human adipocyte proteome from subcutaneous abdominal adipose samples from lean examinees identified three GPXs: GPX1, -4, and probable GPX8 [160]. It is also notable that plasma *GPX3* gene was characterized by the most profound up-regulation (33-fold) during adipogenesis among all genes of enzymes studied. In contrast, GPX1 was characterized only by 7-fold increase of expression [161]. A detailed study using bovine intramuscular preadipocytes demonstrated that adipogenesis is associated with up-regulation of GPX4 expression that may be mediated through C/EBPδ signaling [162]. Lipid accumulation in 3T3-L1 preadipocytes was also associated with increased GPX, as well as SOD, activity [163]. It is notable that GPX3 was found to be a direct estrogen receptor α target gene in WAT that may at least partially mediate the effects of estrogen in reduction of adipose tissue mass [164].

Activity of GPX in adipose tissue was found to be depot-specific. Particularly, SAT GPX activity in lean controls was lower than that in VAT, whereas in obese patients with adverse metabolic profile SAT GPX activity exceeded not only normal-weight values but also BMI-matched GPX activity in VAT [165]. Adipose tissue *GPX3* gene expression was also higher in SAT depots as compared to VAT [166]. It is also notable that SAT GPX3 expression was shown to be higher as compared to omental depot in lean subjects, whereas obesity ameliorated this difference. In turn, weight loss due to bariatric surgery significantly increased SAT GPX3 expression. Although SAT GPX3 expression correlated with adipocyte morphometry, as well as insulin resistance markers, these associations were found to be insignificant after adjustment for BMI, age, and gender [167]. In contrast, SAT GPX activity was found to be increased in patients with both obesity and DM2 when compared to obese or diabetic subjects, as well as metabolically healthy controls [168].

Several investigations have also been performed to assess the impact of obesity on GPX expression and/or activity. Genetically ob/ob mice were shown to be characterized by reduced mitochondrial and cytosolic GPX activity as compared to lean controls [169]. A detailed study by Kobayashi et al. (2009) demonstrated that obesity in ob/ob mice is associated with a significant decrease of cellular GPX activity (GPX1, -4, and -7), as well as increased glutamate-cysteine ligase, catalytic subunit (γ-GCS), whereas inhibition of GPX activity is associated with impaired insulin signaling in 3T3-L1 adipocytes [170]. In 7-week-old KKAy mice, mRNA expression of GPX, as well as Cu,Zn-SOD and catalase in WAT, was found to be significantly reduced as compared to C57BL/6 mice, whereas no differences were observed at the age of 13 weeks [171]. It is also notable that despite a significant increase in serum and kidney GPX activity in obese OLETF rats, adipose tissue GPX activity was reduced. Moreover, both troglitazone (TZD) treatment and obesity decreased GPX1 and GPX3 protein levels in adipose tissue, being indicative of low contribution of adipose tissue GPX to serum GPX activity [172]. In contrast, inguinal WAT GPX activity in obese Zucker rats did not differ significantly from lean animals [173]. HFD feeding was also shown to reduce *GPX3* gene expression in retroperitoneal adipose tissue of rats [174]. Obesity-induced reduction of adipose tissue GPX3 expression was also shown to play a significant role in proinflammatory reaction through induction of both local and systemic oxidative stress [175]. These findings were generally confirmed in human studies. Particularly, high caloric intake through consumption of fast food resulted in a significant down-regulation of adipocyte GPX3 expression, whereas GPX1 expression was positively associated with increased caloric intake [176].

PPARγ deficient mice were characterized by resistance to insulin resistance, as well as increased antioxidant enzyme activity, including GPX in epidydimal adipose tissue, but not skeletal muscle or liver [177].

Although the effect of obesity on tissue GPX was clearly demonstrated, GPX is not only a passive marker of excessive adiposity. Particularly, altered GPX protein and activity may play a significant role in pathogenesis of obesity and associated metabolic disturbances. A pioneer study by McClung et al. (2004) demonstrated that overexpression of GPX1 in mice fed a normal diet resulted in development of increased body weight and excessive adiposity, insulin resistance, and hyperleptinemia. These changes were associated with a significant reduction in insulin-induced phosphorylation of insulin receptor β-subunit in liver and Akt in liver and muscle, being indicative of impairment of redox regulation of insulin signal transduction [178]. These findings are generally in agreement with the later observation of hyperinsulinemia in GPX1-overexpressing mice characterized by increased pancreatic and duodenal homeobox 1 (PDX1) and reduced uncoupling protein 2 (UCP2) levels in pancreas [179]. Correspondingly, *Gpx1*^−/−^ mice fed a HFD were characterized by significantly lower rate of hepatic steatosis and insulin resistance [180]. Gpx1-deficient epididymal adipocytes were also found to have lower diameter despite normal differentiation status (PPARγ, AP2, and C/EBP), being in agreement with the overall resistance of *Gpx1*^−/−^ mice to diet-induced obesity [181]. Moreover, similar effects were observed in *Gpx1*^−/−^ × *Cat*^−/−^ mice due to reduced proinflammatory signal expression and increased WAT browning [182]. At the same time, a high fat and sucrose diet in mice with GPX haploinsufficiency (Gpx1^+/−^) did not aggravate adiposity, but resulted in an adverse metabolic profile characterized by more pronounced glucose intolerance, dyslipidemia, hepatic steatosis, and cardiac fibrosis [183]. Dietary Se deficiency and(or) diet restriction partially alleviated the negative impacts of GPX1 overexpression on lipid and glucose metabolism in male mice [184,185]. In contrast, the GPX mimetic could restore partially the impaired glucose-stimulated insulin secretion and related signaling in the GPX1^−/−^ mice [186].

### 5.4. Thioredoxin Reductases (TXNRDs)

Certain studies demonstrated the role of TXNRDs in modulation of adipogenesis [187]. Adipocyte differentiation is associated with increased activity of both Txnrd1 and Txnrd2 along with Gpx4, whereas antiadipogenic agents decreased Txnrd activation in preadipocytes, but not in mature adipocytes [188]. Correspondingly, TXNRDs transcription in human SAT is significantly associated with lipogenesis and insulin resistance in adipocytes [99]. Moreover, antiadipogenic effect of N-γ-(l-glutamyl)-l-selenomethionine (Glu-SeMet) in glucose-exposed *Caenorhabditis elegans* was dependent on stearoyl-CoA desaturases (SCDs) and Txnrd1 [189]. The particular mechanisms linking TXNRD activity and adipogenesis may include modulation of adipocyte cell cycle. Particularly, Txnrd-deficient fibroblasts were characterized by promotion of cell cycle (p27 and p53 down-regulation), up-regulation of PPARγ, and Akt activation [190].

These effects may be mediated through the influence of TXNRD on protein redox homeostasis and oxidative stress that are known to have significant impact on adipogenesis [191]. Reduced Txnrd1 expression in adipose tissue is associated with increased iNOS activity and S-nitrosylation of adipocyte proteins in response to high fat feeding in mice that may provide a link to obesity-associated metabolic disturbances [192]. It is also important to note the effect of TXNRD in obesity and adipogenesis may be at least partially mediated through modulation of thioredoxin [193] and thioredoxin interacting protein (TXNIP) [194] levels, being involved in redox regulation of adipogenesis.

Clinical studies have also demonstrated a significant association between adipose tissue TXNRD expression/activity, antioxidant regulating pathways, and excessive adiposity. Particularly, in subcutaneous adipocytes from nondiabetic subjects treated with palmitate TXNRD1, as well as TXNRD1-coregulated genes (thioredoxin, thioredoxin domain-containing proteins 9 and 17), correlated significantly with percentage of body fat, corresponding to the observed Nrf2 up-regulation [195]. At the same time, a diet rich in saturated fatty acids induced a significant postprandial decrease in TXNRD1 expression, as well as certain other antioxidant enzymes in adipose tissue, whereas Keap1 was characterized by a postprandial increase [196].

### 5.5. Deiodinases (DIOs)

Consistent with a tight interplay between WAT and thyroid [197], adipose tissue was found to be a source of DIO expression. Particularly, DIO2 mRNA expression and activity was detected in human preadipocytes [198]. Total WAT Dio1 and Dio2 mRNA expression corresponds to 1% of hepatic Dio1 and 7% of brown adipose tissue (BAT) Dio2 mRNA expressions [199]. Adipose tissue-specific DIOs were shown to differentially respond to thyroid status. Particularly, thyroid hormones significantly down-regulated adipocyte Dio2 but not Dio1 activity [199]. In addition, adipose-specific DIO mRNA expression is associated with adipocyte proliferation and differentiation [200].

Adipose tissue remodeling in obesity has a significant impact on adipocyte DIO expression and activity. Particularly, DIO1, but not DIO2 or DIO3, mRNA expression and activity was up-regulated in subcutaneous and omental adipose tissue in obesity, being positively correlated with leptin and inversely associated with SCD1 expression [201]. Leptin injection and caloric restriction were shown to increase and decrease DIO1 activity in adipose tissue, respectively [202]. Bradley et al. (2018) revealed higher DIO2 expression in both subcutaneous and visceral WAT depots of obese subjects. Moreover, increased DIO2 expression was tightly associated with reduced lipid catabolism and mitochondrial dysfunction, but not insulin resistance of proinflammatory cytokine synthesis [203]. Another study revealed a significant decrease in DIO2 expression in visceral and in subcutaneous adipose tissues of morbidly obese subjects [204]. Moreover, SAT DIO2 expression negatively correlated with blood pressure, triglyceride levels, and HOMA-IR values in obese subjects with MetS [205].

Experimental studies also demonstrated the potential role of DIO in obesity. Specifically, *Dio2*-KO was shown to increase susceptibility to HFD-induced obesity with both adipose tissue and ectopic fat accumulation, as well as insulin resistance [206]. However, Castillo et al. (2011) revealed increased proneness to obesity in *Dio2*-KO mice only at thermoneutrality (30 °C), but not ambient room temperature (22 °C) [207]. However, neonatal hepatocyte-specific *Dio2*-KO impaired response to HFD, resulting in alteration of lipid metabolism and PPARy expression, as well as lower rate of obesity and hepatic steatosis [208]. It is also notable, that the role of DIO in obesity may be also mediated through its contribution to regulation of thermogenesis in BAT [209].

The existing data also demonstrate a tight interplay between leptin signaling and DIO activity, being indicative of the potential involvement of the latter into central mechanisms of energy homeostasis regulation [210,211,212].

In view of the key role of iodine in thyroid hormone synthesis and metabolism, the reviewed data may be indicative of the potential involvement of iodine in adipokine regulation and obesity [213]. Particularly, iodine as a constituent of iodine-enriched yolk significantly reduced adipocyte hypertrophy associated with down-regulation of PPARγ, adipogenin, and leptin expressions [214]. Taken together, these findings support the role of Se and iodine interplay in insulin resistance and DM2 both pathogenetically associated with obesity [215]. However, the particular Se-iodine interaction at adipose tissue level is still to be elucidated.

### 5.6. Other Selenoproteins

Single studies have demonstrated involvement of other selenoproteins in adipose tissue physiology. Specifically, selenoprotein 15 kDa (Selenof) was shown to be associated with lipid droplet formation in murine adipocytes [216]. Selenoprotein N (SELENON) was also shown to be involved in adipose tissue-derived stem cell survival and self-renewal [217]. Generally, involvement of certain selenoproteins in regulation of adipocyte functioning was demonstrated in knockout models. Particularly, selenoprotein V gene (*Selenov*)-KO C57BL/6J mice were characterized by increased body weight, as well as adipose tissue accumulation accompanied by increased *Fasn*, *Acaca*, *Dgat1*, and *Lpl* gene expression, in WAT. Moreover, the genes of lipid catabolism and anabolism (accumulation) were down- and up-regulated in *Selenov*^−/−^ mice, respectively, being indicative of the protective effect of this selenoprotein in diet-induced obesity [218]. In addition, *Selenov*-KO significantly modulated selenoprotein response to HFD in mice, resulting in altered Txnrd1 expression in WAT, as well as other tissues [219]. Similarly, selenoprotein M gene (*Selenom*)-KO was associated with increased weight gain and adipose tissue accumulation at least partially associated with hypothalamic leptin resistance, being indicative of the potential involvement of this selenoprotein into central regulation of energy metabolism [220]. Suppression of ethanolamine phosphotransferase 1 (EPT1, selenoprotein I) was also shown to underlie proadipogenic signaling of microRNA-16–5p characterized by up-regulation of PPARγ, SREBP1, C/EBPα, and ACC1 mRNA expressions and protein levels [221].

Methionine sulfoxide reductases (MSRs) may be also involved in development of obesity and/or its associated metabolic disturbances. Particularly, diet-induced obesity in HFD-fed (45% calories from fat) reduced both MsrA and MsrB (predominantly Msrb1) activities, as well as their protein abundances in VAT, but not SAT [222]. Moreover, Novoselov et al. (2010) demonstrated that dietary regulation of MsrB1 activity with Se is improved under caloric restriction [223].

Dietary obesity was also shown to affect selenoprotein metabolism in non-adipose tissues. HFD-feeding resulted in obesity and insulin resistance, as well as significantly reduced muscle selenoprotein W (Selenow) content, although Selenow deficiency was not associated with antioxidant levels, being indicative of lack of protective effect of Selenow in obesity [224]. Prenatal HFD exposure also resulted in a significant increase in Selenow and Selenom expression in mammary glands in prepubertal rats [225]. HFD significantly reduced lymphocyte selenoprotein expression (except Gpx1, Gpx2, Selenot, and Selenom) and this effect was aggravated by low-Se intake. At the same time, low Se intake significantly potentiated proinflammatory effect of HFD through up-regulation of TNFα, NF-κB, IL1, IL6, IL8, and IL9 expressions [226]. It has been demonstrated that obesity up-regulates hepatic expressions of MSRB1, SELENON, -P, and -W, as well as GPX4 in diabetic patients by 33–50%, as compared to non-obese examinees [227]. However, it has been proposed that diet-induced alterations of selenoproteins in other tissues may underlie only obesity-associated metabolic disturbances, rather than play a role in adipocyte dysfunction. For example, selenoprotein gene polymorphisms may at least partially mediate the association between obesity and increased risk of colon and rectal cancer [228].

### 5.7. Proteins Involved in Selenoprotein Synthesis and Degradation

Proteins involved in regulation of selenoprotein synthesis and degradation also play a significant role in adipose tissue physiology and pathology. Selenocysteine insertion sequence-binding protein 2 (SECISBP2) plays a significant role in selenoprotein synthesis [229]. Although direct data on the specific role of SECISBP2 in adipocyte physiology are not available, one may posit its involvement in regulation of adipocyte functions through modulation of selenoprotein synthesis. A case report series has demonstrated that SECISBP2 mutations are associated with increased body fat mass, although metabolic profile including insulin resistance was not affected [230]. A novel homozygous *SECISBP2* gene mutation in a Turkish boy was also associated with obesity, as well as impaired thyroid metabolism [231]. It is also noteworthy that suppression of SECISBP2 in adipose tissue macrophages induces M1 phenotype activation, proinflammatory signaling with subsequent adipose tissue infiltration and inflammation, as well as its hypertrophy and insulin resistance [232]. It is proposed that impaired SECISBP2 activity may significantly alter selenoprotein machinery in adipose tissue leading to its dysregulation.

Selenocysteine lyase (Scly) is also involved in intracellular Se metabolism and recirculation during selenoprotein degradation [233]. Seale et al. (2019) reported that *Scly*^−/−^ mice maintained at Se-deficient diet were characterized by increased body weight, hyperlipidemia, and insulin resistance, but impaired retinoid X receptor (RXR) signaling [234,235]. These findings generally correspond to the earlier findings of the authors demonstrating that Se-deficiency in *Scly*^−/−^ mice resulted in obesity, hyperleptinemia, insulin resistance, and reduced hepatic GPX1 and Selenos levels in parallel with decreased circulating Selenop [236].

A genome-wide association study in high fat and cholesterol diet-fed mice presented a significant inverse correlation (r = −0.68; *p* < 0.001) between epididymal adipose tissue selenophosphate synthetase 2 gene (*Sephs2*) expression and BF% [237], being indicative of the potential role of impaired Sephs2 in obesity pathogenesis.

Taken together, even these single findings demonstrate that maintenance of selenoprotein synthesis (SECISBP2 and Sephs2) and degradation (Scly) in adipose tissue is essential for proper metabolic control of adipogenesis.

## 6. Selenium and Brown Adipose Tissue (BAT)

Brown adipose tissue was shown to play a significant role in energy metabolism thus being considered as the potential target for anti-obesity strategies [238]. DIO2 is the one of the most abundant selenoproteins expressed in BAT [199]. In turn, thyroid hormone signaling modulated by DIO2 activity plays a significant role in BAT thermogenesis through up-regulation of UCP1 expression [209]. Central leptin administration also resulted in a significant increase in DIO2 expression in BAT, being associated with increased UCP-1 mRNA expression [239]. Moreover, formation of brite adipocytes in cold-exposed rats is associated with up-regulation of *Dio2* gene expression, as well [240].

However, the existing data on the association between Se status and BAT physiology are also insufficient. Earlier data demonstrate that Se deficiency is associated with impaired thyroid hormone metabolism and reduced uncoupling protein levels [241] that may provide a link between Se deficiency, lower thermogenesis, and higher susceptibility to obesity. It is also notable that BAT GPX activity is also increased during cold adaptation in order to overcome excessive ROS production under these conditions [242], being in agreement with data on high level of GPX3 expression in BAT [175].

## 7. Se in Central Control of Food Intake

Although being not directly related to adipose tissue metabolism, impaired hypothalamic control of feeding behavior though modulation of the balance between orexigenic and anorexigenic signals plays a significant role in pathogenesis of obesity [243]. Therefore, the existing data on the role of Se in satiety center functioning will be briefly reviewed herein.

Recent data demonstrate that Se and selenoproteins are involved in hypothalamic leptin signaling [244]. This suggestion is supported by the observation of high rate of selenoprotein transcription in Agouti related protein (Agrp) and Proopiomelanocortin (POMC)-expressing neurons [245]. Tight interrelationship between leptin signaling and Selenom was demonstrated by Gong et al. (2019). Particularly, leptin increased hypothalamic Selenom expression, whereas Selenom deficiency significantly impaired leptin-induced STAT3 phosphorylation and subsequent cytosolic calcium responses [246]. Selenocysteine-tRNA gene (*Trsp*)-KO using rat-insulin-promoter-driven-Cre (RIP-Cre) in mice resulted in a significant suppression of leptin signaling and reduction of POMC-positive neurons in hypothalamus, whereas Nrf2 signaling reversed both hypothalamic oxidative stress and leptin resistance [247]. Another selenoprotein, GPX4, was also shown to be involved in hypothalamic energy metabolism control. Knockout of GPX4 in Agrp, but not POMC neurons resulted in increased weight gain and adipose tissue mass [248].

Direct impact of Se treatment on signals food control and satiety center was demonstrated. Particularly, p-chloro-diphenyl diselenide was shown to possess anorexigenic effects through modulation of food behavior, satiety signals, and diet palatability [249]. These effects may be at least partially mediated by reduced hypothalamic orexin production [250]. The impact of Se on appetite regulation was also demonstrated in poultry. Specifically, depending on the dose of dietary Se intake resulted in increased neuropeptide Y gene (*NPY*) expression (3.0 mg/kg) or increased leptin receptor mRNA levels (5.0 mg/kg) in hypothalamus, although both doses may be considered as supraphysiological [251].

Several studies have indicated that central effects of selenium may be mediated through regulation of DIOs. Hypothalamic and hepatic DIO activity and expression were also shown to be differentially regulated in obesity-prone and obesity-resistant rats under HFD-feeding [252]. Finally, *Dio3*-KO mice were characterized by reduced adiposity, as well as aberrant expression of leptin-melanocortin system genes (Agrp, Npy, Pomc, Mc4r), in parallel with leptin resistance [253].

Several studies have demonstrated a significant impact of Se on leptin levels. Specifically, consumption of Se-rich (480 µg/L) hot spring water in rats resulted in a nearly twofold decrease in serum leptin levels [254]. However, this effect may be mediated by both reduced adipose tissue mass and reduction of leptin resistance. Similar effects were observed in prenatal modulation of Se status. Feeding pregnant and lactating dams with Se-deficient chow (0.01 µg/g diet) resulted in growth retardation, as well as increased leptin levels in offspring at weaning (21 day old), whereas dietary Se exposure (0.5 µg/g diet as sodium selenite) resulted in obesity, insulin resistance, inflammation, as well as low circulating leptin levels [255]. Se deficiency in weanling rats fed for 4–10 weeks with low-Se diet was associated with a nearly 50% decrease in circulating leptin levels, being associated with a decrease in corticosterone levels in adrenocorticotropic hormone-stimulated rats [256].

Taken together, these findings clearly demonstrate interference between Se metabolism and hypothalamic food control that may be mediated through the role of selenoproteins in modulation of redox homeostasis (GPXs and TXNRDs) and ERS (SELENOM) in hypothalamic neurons [244].

## 8. Conclusions

**Figure 4:**
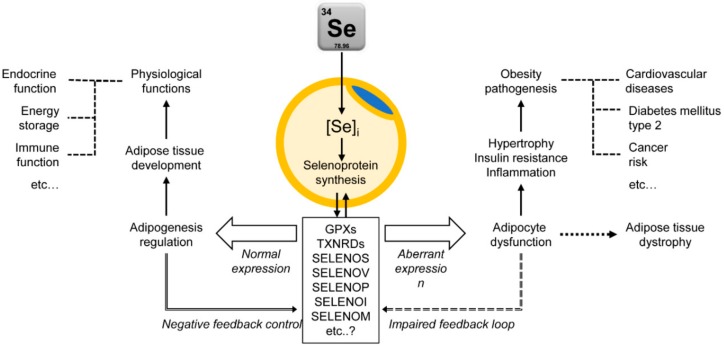
The proposed role of Se in adipose tissue physiology and obesity pathogenesis. Adipocyte intracellular Se levels [Se]_i_ are associated with activity of selenoprotein metabolism machinery (SECISBP2, Sephs2, and Scly) regulating adipose tissue selenoprotein expression. Endoplasmic reticulum resident selenoproteins (SELENOS, SELENOV, etc.) together with mitochondrial and cytosolic GPXs and TXNRDs were shown to interfere with adipocyte development and functioning. Adequate Se supply (including its transport with SELENOP), as well as normal selenoprotein expression, is essential for regulation of adipogenesis and physiological development of adipose tissue, leading to its physiological functioning, including energy storage, endocrine, and immune functions. In turn, both low and increased expression of adipocyte selenoproteins may result in adipose tissue dysfunction contributing to adipocyte hypertrophy or dystrophy, insulin resistance, and adipose tissue inflammation. It is hypothesized that impairment of negative feedback mechanism may partially underlie the association between aberrant selenoprotein expression and adipocyte dysfunction. However, the particular relationship between Se intake, systemic Se levels, adipose tissue Se and adipocyte selenoprotein expression is not clear. GPXs, glutathione peroxidases; Scly, selenocysteine lyase; Se, selenium; SECISBP2, selenocysteine insertion sequence-binding protein 2; SELENOI/-M/-P/-S/-V, selenoprotein I, M, P, S, and V; Sephs2, selenophosphate synthetase 2; TXNRDs, thioredoxin reductases.

In conclusion, Se and selenoproteins are essential for adipose tissue development and functioning. Particularly, Se regulates progenitor cell proliferation, adipocyte differentiation, and maturation, as well as cellular functions (lipid accumulation and lipolysis), although the effect may be different among selenate, selenite, and organic Se compounds. In view of the specific functions of selenoproteins, the mechanisms underlying their effects on adipose tissue may be predominantly related to modulation of redox homeostasis and ERS. These mechanisms are implicated into the action of Se in hypothalamic regulation of satiety and food behavior. Thus, impaired selenoprotein synthesis may underlie adipocyte dysfunction leading to various diseases including development of obesity, as demonstrated in knockout models. At the same time, Se overexposure may also impair selenoprotein expression and activity [257] and thus impair adipocyte functioning. Therefore, both Se deficiency and overload may alter selenoproteome [258] and lead to adipose tissue dysfunction (Figure 4).

Human studies demonstrated significant alterations in Se status in patients with obesity, although some of the reviewed results are contradictory, being indicative of both increased or decreased Se and biomarker levels. Such discrepancies may occur from different environmental exposure levels, as well as Se species in the studied populations. It is also rather problematic to dissect obesity from insulin resistance together with DM2, known to be associated with increased Se exposure. In addition, the particular markers of Se status currently used may affect the outcome of such studies. Serum/plasma speciation analysis currently lacking in obesity research using hyphenated techniques [259,260] may be significantly helpful to overcome the issue due to dissection of particular Se distribution. Moreover, Se speciation in adipose tissue could provide a significant contribution to understanding of local patterns of Se handling in adipocyte under a variety of pro/anti-adipogenic signals, although the particular techniques are still to be developed in view of high lipid/low protein content of adipose tissue.

Taken together, these issues currently do not suggest the utility of Se supplementation in obesity and raise some concerns about its safety. Despite the proposed protective effect of Se in MetS [261], its administration without a prior assessment of the Se status of the individual may result in overwhelming the benefits by hazardous effects [262].

However, recent data from both human and laboratory studies on the role of Se and selenoproteins in obesity and adipose tissue function are highly heterogenous. The existing inconsistencies may occur from differences in dietary Se intake, ethnic, and physiologic characteristics of the studied populations, as well as from variability of Se status markers used. In turn, laboratory studies should consider for the dose, species, as well as the regimen of treatment in order to model physiologically-relevant conditions. Functional heterogeneity of adipose tissue depots [263] in the earlier studies may also mediate different associations between the used obesity models and selenium metabolism. It is also notable that extrapolation of the laboratory data to human should be undertaken with caution due to differences in selenoprotein metabolism [4].

Further human and laboratory studies are required to highlight the association between Se intake and obesity, as well as particular mechanisms of Se and selenoprotein action in adipose tissue underlying these effects.

## Supplementary Materials

The following are available online at https://www.mdpi.com/2218-273X/10/4/658/s1, Figure S1: Mean difference in individual studies and summary standardized mean difference (SMD) of selenium (Se) levels (**A**) and glutathione peroxidase (GPX) activity (**B**) in patients with obesity compared with controls, children population, Table S1: A summary of studies investigating the association between blood selenium levels in relation to increased BMI or obesity, Table S2: A review of studies demonstrating the relationship between BMI and obesity with blood GPX activity, Table S3: A brief summary of studies demonstrating the association between selenoprotein P (SELENOP) levels and obesity.
