# Supplementary material for: Selenium and Selenoproteins in Adipose Tissue Physiology and Obesity
Source: Biomolecules. 2020 Apr 24;10(4):658. doi: 10.3390/biom10040658 (PMC7225961; doi:10.3390/biom10040658)
Supplement: Supplementary file 1 [file biomolecules-10-00658-s001.zip › Table_S1.pdf]

Table S1. A summary of studies investigating the association between blood selenium levels in relation to increased BMI or obesity.

Population	Study design	Year	N (M/F)	Sex	Age, y	Specimen	Se levels	Statistics	Adjustments factors	Ref.
Health survey evaluation in China	Cross-sectional	2013	1466 (939/527)	Both (M/F)	22–81	Blood (μg/L)	Median (25-75)<br>94 (87–103) NGg (n=1356) vs 100 (90–110) HGg (n=110), p<0.001<br>97 (89–106) DLg (n=873) vs 92 (84–100) NLg (n=593), p<0.001	ORs (95%CI) for Q1-Q4<br>HGg/NGg: 1.0, 1.3 (0.65–2.7), 1.7 (0.84–3.3), 2.9 (1.5–5.5), p=0.001<br>DLg/NLg: 1.0, 1.6 (1.2–2.2), 2.1 (1.5–2.8), 2.9 (2.1–4.0), p<0.001	Adjusted for potential confounders including age, gender, body mass index, and mineral elements	[39]
NHANES III 1988-1994	Cross-sectional	1988-1994	13 289 (6440/6849)	Both (M/F)	44.2±0.4	Blood (ng/mL)	Mean by quartile of %BF:<br>M 127.6 Q1(≤20.1), 127.5 Q2 (20.1–24.2), 127.4 Q3 (24.2–27.9), 126.2 Q4 (27.9–49.8)<br>F 125.4 Q1(≤ 29.7), 126.1Q2 (29.7–35.4), 124.7Q3 (35.4–40.7), 121.9 Q4 (40.7–59.4)	Difference (95%CI) by quartiles of %BF (Q4 and Q1)<br>M −1.7 (−4.2, 0.7) (nonsignificant)<br>F −4.5 (−7.0, −1.9)	Adjusted for age (and race/ethnicity, education (<12 years, ≥12 years), postmenopausal status for women, smoking, serum cotinine and sedentary lifestyle, diabetes, C-reactive protein (<0.3, ≥0.3 to <1, ≥1 mg/dL), systolic blood pressure, antihypertensive medication, total cholesterol, triglycerides, and HDL-cholesterol	[30]
							Mean by quartile of BMI<br>M 128.8 Q1 (≤23.5), 127.2 Q2 (23.6–25.9), 127.2Q3 (26.0–29.0), 125.6 Q4 (≥29.1)<br>F 125.6 Q1 (≤21.9), 125.6 Q2 (22.0–25.1), 124.0 Q3 (25.2–29.9), 123.2 Q4 (≥30.0)	Difference (95%CI) by quartiles of BMI (Q4 and Q1)<br>M/F −4.0 (−5.5, −1.6)		
Health survey evaluation in State of Kuwait	Case-control (Non-Ob=44, Ob=66)	2009-2010	110	F	30.75± 9.40 (Non-Ob) - 28.5± 7.0 (Ob)	Blood (μg/L)	Mean±SD<br>101.14 ± 11.34 (Non-Ob) vs 86.08 ± 14.10 (Ob)			[31]
Health survey	Case-control	2012	573		8 - 13	Blood (μg/L)	Mean±SD	BMI: OR = 1.5031 (1.3828 - 1.6338)		[32]

evaluation in Spain							64.6 ± 16.8 (Ob) vs 75.3 ± 12.2 (Non-Ob)<br>Se intake (µg/k)<br>1.99 ± 0.62 (Ob) vs 2.73 ± 0.88 (Non-Ob)	Se intake: OR = 0.9862 (0.9775 - 0.9949)		
Health survey evaluation in Poland	Case-control ((Non-Ob =40, Ob=40)	2015	80 (40/40)	Both (M/F)	6-17/13(3–18)	Blood (µg/L)<br>Urine (µg/L)	Mean±SD<br>Blood: 111.1 ± 9.5, 102.3 ± 7.9 (Non-Ob) vs 82.8 ± 10.3, 80.4±8.2 (Ob)	OR=0.74 (0.62–0.88)		[33]
							Urine: 60.3 ± 11.5, 55.9 ± 9.4 (Non-ob) vs 36.7 ± 5.6, 36.0 ± 7.5 (Ob)	OR=0.60 (0.43–0.83)		
Health survey evaluation in UK	Prospective	2014	437 (78/358)	Both (M/F)	45.9 ± 11.2	Blood (µmol/L)	Median (IQR)<br>1.02 (0.90–1.15) (preoperatively) vs 0.91 (0.82–1.14) (36 months after bariatric surgery)			[34]
Health survey evaluation in France	Cross-sectional	2007-2010	222 (56/166)	Both (M/F)	40.5 ± 12.6	Blood (µmol/L)		Prevalence of deficiency % (n/N)<br>1.8 (1/56) for men, 3.6 (6/166) for women	No adjustment	[35]
SU.VI.MAX Study 1994–1995	Cross-sectional	1994–1995	3128 (1307/1821)	Both (M/F)	35–60/M 52.1± 4.8,<br>F 47.2±6.7	Blood (mmol/L)	Mean±SD (BMI)<br><br>M 1.13 ± 0.19 (<25), 1.13 ± 0.2 (25–30), 1.18 ± 0.23 (>30)	P (difference means)= 0.15, P trends=0.06 (BMI) R^2adj= 0.0232, b=0.02258 0.00582, SE= 0.00582, p=0.001 (Cholesterol, log-transformed)		[36]
							F 1.08±0.19 (<25), 1.11±0.19 (25–30), 1.08±0.19 (>30)	P (difference means)= 0.08, P trends=0.96 (BMI) R^2adj= 0.0350, b=0.01792, SE= 0.00456, p<0.001 (Cholesterol, log-transformed)		
			399			Blood	Mean±SD (BMI)			[37]

ATTICA Study 2001 –2002	Cross-sectional	2001 – 2002	(236/163)	Both (M/F)	18–89/38.56±11.4	(ng/mL-1)	M 86.7622.0 (18–24.99), 80.96±19.9 (25–29.99), 79.46±17.0 (>30), p=0.097	b±SE -0.782±0.371, p=0.036		
							F 86.4624.3 (18–24.99), 86.7623.0 (25–29.99), 79.46±17.0 (>30), p=0.736			
Health survey evaluation in Northern Taiwan	Case-control (Q1 n=213, Q2 n=211, Q3 n=212, Q4 n=211)	2007-2014	847	Both (M/F)	62.7±10.4 (Q1)-65.2±9.6 (Q4)	Blood (µg/L)	Mean (interquartile cut-off values) 88.2±21.2 (71.4, 86.8, and 104.5)	ORs (95%CI) for Q1-Q4 DM2: 1.0, 1.24 (0.78-1.98), 1.90 (1.22-2.97), 5.11 (3.27-8.00)	Adjusted for age, gender, current smoking, current drinking, and physical activity	[40]
							Mean±SD by quartile of Se level, DM2 prevalence 62.0±6.8 Q1 (<71.4 µg/L), DM2=22.7% 79.0±4.6 Q2 (71.4–86.7 µg/L), DM2=25.1% 95.6±5.4 Q3 (86.8–104.5 µg/L), DM2=36.3% 116.3±9.1 Q4 (>104.5 µg/L), DM2=59.2%	DM2: OR=1.0, 0.69 (0.37-1.27), 1.57 (0.91-2.70), 3.79 (2.17-6.32)	Adjusted for age, gender, current smoking, current drinking, physical activity, WC, and HOMA-IR	
Health survey evaluation in Malaysia	Case-control (DM2=82, Controls=82)	2017	164 (78/86)	Both (M/F)	35-55/ DM2 48.60±6.6, Controls 44.62(6.64)	Blood (µg/L)	Mean (95% CI) 90.46 (85.97-94.94) (DM2) vs 88.17 (83.69,92.66) (Controls)	ORs (95%CI) for T1-T3 of Se BMI: 25.79 (24.14–27.45) (DM2) vs 25.87 (24.48–27.26) (Controls) (T1), 28.20 (26.42–29.98) (DM2) vs 26.16 (24.87–27.44) (Controls) (T3) Body fat, %: 29.41 (27.33–31.49) (DM2) vs	Adjusted for age, gender	[41]

								31.23 (29.04–33.43) (Controls) (T1), 32.51 (30.27–34.74) (DM2) vs 31.77 (29.74–33.80) (Controls) (T3)		
Health survey evaluation in Japan	Follow-up	2008-2012	76 (42/34)	Both (M/F)	51.9 ± 10.5	Blood	Mean±SD Serum Se (μg/L) 157.9 ± 21.7 (Baseline) vs 205.0 ± 22.1 (4 year), p=0.075	r=0.329, p=0.029 (WC) r=0.187, p=0.225 (BMI)		[42]
							SELENOP (μg/mL) 2.51 ± 0.52 (Baseline) vs 3.81 ± 0.60 (4 year), p<0.001	r=−0.023, p=0.844 (WC) r=−0.042, p=0.721 (BMI)		
							GPX3 activity (U/L) 190.4 ± 68.5 (Baseline) vs 238.3 ± 55.2 (4 year), p<0.001			
IMMIDIET study	Cross-sectional	2012	1 902 (942/ 960)	Both (M/F)	M Non MS 46.2±7.9 vs MS 49.1±7.5, F Non MS 43.9±7.8 vs MS 49.4±7.2	Blood (mmoll^{-1})	Mean (SD) M 1.24 (0.23) (Non MS) vs 1.24 (0.23) (MS), p=0.84	OR (95%CI) M OR=0.97 (0.81-1.16), p= 0.75	Adjusted for age, country group, social status, physical activity, energy intake, alcohol consumption and smoking.	[43]
							F 1.21 (0.23) (Non MS) vs 1.32 (0.28) (MS), p=0.0002	F OR=1.42 (1.15-1.77), p=0.001		
								F OR=1.33 (1.06-1.67), p=0.01	Adjusted for age, country group, social status, physical activity, energy intake, alcohol consumption and smoking, for menopausal status, uses of oral contraceptive pills or hormonal replacement therapy	

Health survey evaluation in Taiwan 2007-2017	Case-control Q1 n=292, Q2 n=290, Q3 n=292, Q4 n=291	2007-2017	1165 (418/747)	Both (M/F)	65.8 ± 10.3 Q1, 65.9 ± 9.7 Q2, 66.7 ± 9.6 Q3, 64.9 ± 10.3 Q4	Blood (µg/L)	Mean ± SD by quartile of Se level<br>65.13 ± 7.81 Q1 (≤76.0 µg/L),<br>85.16 ± 5.19 Q2 (76.1–94.0 µg/L),<br>104.46 ± 5.59 Q3 (94.1–113.7 µg/L),<br>130.66 ± 14.82 Q4 (>113.7)	ORs (95%CI) for Q1-Q4 (MS)<br>M OR=1.0, 1.62 (0.79–3.31), 1.94 (0.99–3.82), 5.33 (2.94–9.66), p<0.001	Adjusted for age, current smoking status, current drinking status, physical activity, BMI	[44]
								F OR=1.0, 1.03 (0.64–1.65), 2.10 (1.25–3.52), 2.38 (1.18–4.83), p=0.015		
Health survey evaluation in Indonesi a	Case-control Ob n = 26, (Ob + 1 component n = 28, MS n = 24	2013	78	M	39.7 ± 7.7 (Ob), 38.4 ± 5.6 (Ob + 1 component), 36.4 ± 5.8 (MS)	Blood	Mean ± SD (Range)<br>Serum Se, ng/mL<br>95.8 ± 20.2 (62.0–163.0) (Ob)<br>102.2 ± 20.7 (63.0–162.0) (Ob + 1 component)<br>105.6 ± 22.0 (54.0–143.0) (MS)	HDL: r=0.390, p<0.05 (Ob), r=0.105, p>0.05 (Ob + 1 component), r=0.013, p>0.05 (MS)<br>FABP4: r=0.474, p<0.05 Ob), r=0.023, p>0.05 (Ob + 1 component), r=−0.083, p>0.05 (MS)		[45]
							GPX activity (Δmmol NADPH mL⁻¹ min⁻¹)<br>166 ± 21 (116–215) (Ob)<br>163 ± 26 (110–225) (Ob + 1 component)<br>172 ± 20 (140–210) (MS)	HDL: r=0.075, p>0.05 (Ob) r= 0.413, p<0.05 (Ob + 1 component)<br>r=0.189, p>0.05 (MS)<br>FABP4: r=−0.467, p<0.05 Ob), r=−0.190, p>0.05 (Ob + 1 component), r= −0.077, p>0.05 (MS)		
Health survey evaluation in Brazil	Trial Ob n = 29, Control n = 26	2015	55	F	18-55	Blood	T0 (Baseline)–T1(after 2 mo)<br>Plasma Se (mg/L)<br>87.1 (82-97.7) – 244 (226-278) (Ob) vs 65.7 (61.7-71.3) – 63.2 (60.6-67.7) (Control)	r=0.579 p<0.001 (TNFα)<br>r=0.625, p<0.001 (IL-6)<br>r=0.567, p<0.001 (IL-10)<br>r=0.662, p<0.001 (TLR2)<br>r=0.753, p<0.001 (TLR4)		[48]
							RBC Se (mg/L) 125 (122-143) – 351 (309-480) (Ob)	r=0.564, p<0.001 (TNFα)<br>r=0.647, p<0.001 (IL-6)<br>r=0.437, p=0.001 (IL-10)		

							vs 125 (120-141) – 130.2 (116-146) (Control)	$r=0.561$, $p<0.001$ (TLR2)<br>$r=0.698$, $p<0.001$ (TLR4)		
							SELENOP (ng/mL) 37.7 (16.1-51.9) – 55.5 (37.1-150.6) (Ob) vs 28.5 (12.5-52.1) – 23.6 (16-32.6) (Control)	$r=0.433$, $p=0.003$ (TNFα)<br>$r=0.636$, $p<0.001$ (IL-6)<br>$r=0.369$, $p=0.008$ (IL-10)<br>$r=0.321$, $p=0.037$ (TLR2)<br>$r=0.421$, $p=0.004$ (TLR4)		
							GPX1 activity (U/gHb) 48.7 (37.5-57.6) – 57.2 (45.8-67.5) (Ob) vs 47.2 (41-57.1) – 49.7 (43.5-58.5) (Control)			

MS – metabolic syndrome; BMI – body mass index; BF – body fat; NGg – normal glucose group; NLg – normal lipid group; DLg – dyslipidemia group; HGg – hyperglycemia group; Ob – obesity; DM2 – diabetes mellitus type 2; GPX – glutathione peroxidase; SELENOP – selenoprotein P; RBC – red blood cells; FABP4 – fatty acid binding protein 4; TNFα – tumor necrosis factor α; IL – interleukin; TLR – Toll-like receptor; WC – waist circumference; HOMA-IR – homeostasis model assessment-insulin resistance
