# Supplementary material for: Selenium and Selenoproteins in Adipose Tissue Physiology and Obesity
Source: Biomolecules. 2020 Apr 24;10(4):658. doi: 10.3390/biom10040658 (PMC7225961; doi:10.3390/biom10040658)
Supplement: Supplementary file 1 [file biomolecules-10-00658-s001.zip › Table_S2.pdf]

Table S2. A review of studies demonstrating the relationship between BMI and obesity with blood GPX activity

Population	Study design	Year	N (M/F)	Sex	Age	Specimen	Se levels	Statistics	Adjustments factors	Ref.
Health survey evaluation in Algeria	Case-control Young n=110, old n=95	2011	205	M	20-75	Blood	GPX activity (U/ g Hb)	BMI: r=-0.177, p>0.05 (Young) vs r=-0.252, p>0.05 (Old) R^2=0.15, b (SE) =-0.232 (0.08), p=0.04 BMI and age: R^2=0.28, b (SE)=-0.475 (0.05), p=0.001		[49]
							SOD activity	BMI: r=0.431, p<0.001 (Young) vs r=-0.522, p<0.001 (Old) R^2=0.22, b (SE) = 0.411 (0.05), p=0.001 BMI and age: R^2=0.75, b (SE) =0.751 (0.04), p<0.001		
							Catalase activity	BMI: r=0.227, p>0.05 (Young) vs r=-0.263, p>0.05 (Old) R^2=0.21, b (SE) =0.248 (0.07), p=0.04 BMI and age: R^2=0.34, b (SE) = 0.583 (0.06), p=0.001		
Health survey evaluation in Kuwait	Case-control Control n=50, Ob=250	2002	300 (120/180)	Both (M/F)	20-75	Blood	GPX activity (units/g Hb) Mean ± SD by quartile of BMI 98.4 ± 3.3 Q1 (19 – 25), 95.1 ± 3.6 Q2(30 – 34), 90.0 ± 7.4 Q3 (35 – 39), 84.3 ± 6.7 Q4 (40 – 44), 80.8 ± 7.2 Q5 (45 – 49), 76.3 ± 6.9 Q6(>50)	BMI: r=-0.436, p=0.018		[50]

Health survey evaluation in Iran	Case-control Control n = 79, Owerweight n = 56, Ob n = 25	2014	160	F	20-45/27± 9 vs 35± 8 vs 39± 6	Blood	GPX activity (U/gHb) Mean ± SD 148.7 ± 54.2 (Controls) vs 124.6 ± 48.9 (Overweight) vs 97.6 ± 45.2 (Ob), p=0.005 154.7 ± 62.4 (normal) vs 103.6 ± 52.8 (abdominal obesity), p<0.01	BMI: r=−0.275, p<0.01 R^2=7.2%, b=−0.312, p=0.002	Adjusted for age, physical activity, number of pregnancies, systolic and diastolic blood pressures and nutrient intakes (energy, protein, total fat and carbohydrate intake)	[51]
Health survey evaluation in UK	Case-control Ob n=82, Non-Ob n=155, MS n=161, Non-MS n=76, Control n=135	2008	237 (142/95)	Both (M/F)	55.1±13.3	Blood	Mean ± SD Serum Se (μmol/L) 1.04±0.24 (Ob) vs 1.10±0.23 (Non-Ob); 1.08±0.23 (MS) vs 1.08±0.23 (Non-MS); 1.03±0.03 (Control)			[52]
							GPX activity (U/mL) 0.28±0.09 (Ob) vs 0.33±0.10 (Non-Ob); 0.31±0.11 (MS) vs 0.31±0.08 (Non-MS); 0.36±0.12 (Control)			
Health survey evaluation in South Korea	Case-control WHR ≤ 0.90 n=11, 0.90 < WHR ≤ 0.95 n=32, WHR > 0.95 n=30	2008-2010	73	M	45.1 ± 7.2 - 46.6 ± 8.	Blood	Mean ± SD GPX activity (nmol/min/mL) Mean ± SD by WHR 107.5 ± 37.7 (25.4 ± 3.4) vs 123.4 ± 38.0 (26.5 ± 2.7) vs	r=0.318, p<0.01 (WHR)		[53]

							135.4 ± 35.6 (26.9 ± 2.8), p=0.095			
British NDNS	Cross-sectional	2000–2001	1050 (512/538)	Both (M/F)	41.0 (31.0, 53.0)	Blood	Median (Q1-Q3) by BMI<br>GPX activity (nmol mg Hb^{-1} min^{-1})<br>121.3 (106.0, 145.0) (<25), 121.0 (103.0, 144.0) (25-29), 115.7 (98.4, 135.0) (>30)	Difference (95% CI) by WHR (Q4-Q1)<br>-7.4 (-12.7, -2.0), p<0.05	Adjusted for age	[54]
								Difference (95% CI) by BMI (Q3-Q1)<br>−7.9 (−13.2, −2.7), p<0.05<br>Difference (95% CI) by WC (Q4-Q1)<br>−9.7 (−16.2, −3.2), p<0.05	Adjusted for sex, menopausal status, household income group, educational level group, employment, daily food energy, total fat intake, total cholesterol intake, polyunsaturated-to-saturated fatty acids ratio, carbohydrate intake, starch intake, nonmilk extrinsic sugar intake, protein intake, sodium intake, smoking status, daily cigarette consumption, current drinking status, daily units	
							Plasma Se (mmol/L) by BMI<br>1.09 (0.99, 1.22) (<25), 1.07 (0.97, 1.18) (25-29), 1.08 (0.99, 1.21) (>30)	Difference (95% CI) by BMI (Q3-Q1)<br>-0.03 (-0.06, 0.00), p>0.05<br>Difference (95% CI) by WC (Q4-Q1)<br>-0.03 (-0.07, 0.01), p>0.05		
							RBC Se (mmol/L) by BMI<br>1.63 (1.34, 1.95) (<25) 1.61 (1.40, 1.86), (25-29), 1.62 (1.37, 1.89) (>30)	Difference (95% CI) by WC (Q4-Q1)<br>-0.06 (-0.12, 0.01), p>0.05<br>Difference (95% CI) by WHR (Q4-Q1)<br>0.10 (0.00, 0.20), p<0.05		

									of alcohol, daily physical activity score, vitamin/mineral supplement use, oral contraceptive use, hormone replacement therapy, TC, non-HDL-cholesterol, and TC-to-HDL-cholesterol ratio.	
								Difference (95% CI) by BMI (Q3-Q1) −7.9 (−13.2, −2.7), $p<0.05$	Adjusted for age	
Health survey evaluation in Brazil	Case-control Control n=36, Ob=37	2011	73	F	20-50/ 31.2±7.8 (C), 33.7±7.9 (Ob)	Blood	Mean ± SD GPX activity (U/gHb min)	46.4±19.4 (Obese) vs 36.7±13.6 (Controls), $p<0.05$	b=0.51, t=3.06, p=0.05 (TAG)	[55]
Health survey evaluation in Sri Lanka	Case-control DM2 n=147, PreDM2 n=47, Controls=106	2019	147	Both (M/F)	DM2 47.6 ± 8.3, PreDM2 45. 7 ± 8.8, Controls 44. 2 ± 8.2	Blood	Mean ± SD GPX activity (Ug/Hb per min) 342 ± 287 (DM2+Ob) vs 300 ± 219 (DM2-Ob), 267 ± 147 (Controls+Ob)	BMI: r=0.23, p=0.044 WHR: r=0.20, p=0.043		[56]
							350 ± 291(DM2+COb) vs 300 ± 191 (DM2-COb), 292 ± 243 (PreDM2+COb) vs 271 ± 171 (PreDM2-COb), 272 ± 172	BMI: r=0.24, p=0.047 WHR: r=0.40, p=0.021		

							(Controls+COb), 227 ± 152 (Controls-COb)			
Health survey evaluation in Mexico	Case-control Control n= 28, Ob n=133	2012	161	Both (M/F)	31.7±12.4 (C), 42.8± 10.7 (Ob)	Blood	Mean ± SD<br>GPX activity (ng/dL) 143.5 ± 23.1 (Controls) vs 175.4 ± 26.1 (Overweight) vs 175.2 ± 24.3 (Ob)	Weight: r=0.199, p<0.01<br>TAG/HDL: r=0.228, p<0.01<br>QUICKI index: r=-0.234, p<0.01		[57]
Health survey evaluation in Spain	Case-control HOMA-IR <median n=31, HOMA-IR >median n=29	2009	60 (17/43)	Both (M/F)	42.2 ± 10.7 – 44.9 ± 8.6	Blood	Mean ± SD<br>GPX activity (nmol/min/mL) 19.5 ± 4.6 (HOMA-IR <median) vs 23.5 ± 6.5 (HOMA-IR ≥median)	ΔTAG: r=0.37, p=0.006		[58]
Health survey evaluation in Turkey	Cross-sectional	2007	120 (50/70)	Both (M/F)	8.80 ± 1.26 (Ob), 8.96 ± 1.05 (Controls)	Blood	Mean ± SD<br>GPX activity (U/g Hb) 19.16 ± 2.22 (Ob) vs 23.68 ± 3.58 (Controls), p<0.001		Adjusted for gender or age, height and BMI	[59]
Health survey evaluation in China	Case-control Controls n=30, Ob n=31	2012-2013	61	M	41-47/44.03±1.87	Blood	Mean ± SD<br>GPX activity (U/ml) 860.11±24.81 (Ob) vs 877.40±24.32 (Controls), p<0.01			[60]
Health survey evaluation in Spain	Cross-sectional	2010	68 (38/30)	Both (M/F)	11.6±3.1, 11.1±2.1, 10.2±2.6	Blood	Mean ± SD by quartile of SDS-BMI<br>GPX activity (U^*gHb^{-1}) 89.32 ± 39.94 (<1.33), 139.85 ± 32.48 (2-3), 137.32 ± 78.98 (>3), p=0.002	TC: r=-0.34, p=0.019<br>MDA: r=0.43, p=0.002<br>Vit E: r=-0.32, p=0.028		[61]
Health survey	Case-control	2012	106 (50/56)	Both (M/F)	6–12	Blood	Mean ± SD<br>GPX activity (U/g Hb)	BMI: r=0.17, p=0.28<br>WC: r=-0.05, p=0.86		[62]

evaluation in Tunisia	Control n=52, Ob n=54						**M** 6-8.5 years: 39.77 ± 6.98 (Ob) vs 32.48 ± 7.29 (Controls), 9-12 years: 34.38 ± 8.52 (Ob) vs 33.83 ± 8.9 (Controls)			
							**F** 6-8.5 years: 38.83 ± 7.26 (Ob) vs 34.41 ± 6.22 (Controls), 9-12 years: 36.55 ± 6.48 (Ob) vs 35.64 ± 8.31 (Controls)	BMI: r=-0.03, p=0.87<br>WC: r=-0.23, p=0.39		
Health survey evaluation in Saudi Arabia	Case-control Control n=66, Overweight n=83, Ob n=64	2011-2012	213 (143/70)	Both (M/F)	6-12/9.5 ± 1.5	Blood	Mean ± SD GPX activity (mg/g Hb) 37.3 ± 5.9 (Ob) vs 56.8 ± 3.5 (Overweight) vs 41.4 ± 2.6 (Controls), p=0.001			[63]
Health survey evaluation in Turkey	Case-control Ob-IR n=20, Ob+IR n=22, Controls n=21	2012	63	Both (M/F)	10.27 ± 2.36 (Ob-IR), 11.26 ± 2.52 (Ob+IR), 11.41 ± 2.00 (Controls)	Blood	Mean ± SD GPX activity (U/mL) 0.026 ± 0.011 (Ob+IR) vs 0.046 ± 0.015 (Ob-IR) vs 0.048 ± 0.012 (Controls), p=0.001 (Adolescents) 0.032 ± 0.01 (Ob+IR) vs 0.042 ± 0.01 (Ob-IR) vs 0.048 ± 0.01 (Controls), p=0.005 (Overall)			[64]
Health survey	Trial	2006	66	F	36.7 ± 8.3	Blood	Mean ± SD GPX activity (ng/ml)			[66]

evaluation in Greece							67.5 ± 21.3 (Controls) vs 22.3 ± 9.5 (Ob before) vs 48.9 ± 14.1 (Ob after), p<0.001			
Health survey evaluation in India	Case-control Controls n=20, Ob n=20, Non-Ob+DM2 n=20	2011	60	Unknown	45–65	Blood	Mean ± SD GPX activity (U/mg proteins) 70.9 ± 9.6 (Controls) vs 23.4 ± 3.8 (Ob+DM2 PreIns) vs 26.05 ± 4.03 (Ob+DM2 24w) vs 28.4 ± 6.4 (Ob+DM2 48w); 41.5 ± 3.5 (Non-Ob+DM2 Pre-Insulin) vs 48.7 ±4.8 (Non-Ob+DM2 24w) 51.8 ± 5.4 (Non-Ob+DM2 48w)			[67]
Health survey evaluation in China	Trial Placebo n=45, His n=47	2013	92	F	33–51	Blood	Mean ± SD GPX activity (nmol/ml) 135.3 ± 29.0 (Baseline), 138.1 ± 26.2 (12w) (Controls) vs 136.6 ± 25.4 (baseline), 150.3 ± 28.3 (12w) (Ob)	13.71 [95% CI 9.65, 17.78] His: r=0.324, p=0.009		[69]
Health survey evaluation in China	Case-control Controls n=217, Ob n=235	2012	452	F	43.39±2.57 (C), 44.13±1.73 (Ob)	Blood	Mean ± SD GPX activity (μmol/L) 158.90 ± 36.43 (Ob) vs 167.39 ± 33.01 (Controls), p<0.001	His: b=0.149, p<0.005	Adjusted for age, smoking, alcohol use, menopause and physical activity at work and at leisure, and BMI	[70]

Health survey evaluation in Spain	Case-control Controls n=191, Ob n=193	2014	193 (104/89)	Both (M/F)	3-13/ 8.9±0.1 (C), 8.7±0.1 (Ob)	Blood	Mean ± SD<br>GPX activity (mol/g Hb/min)<br>0.019 ± 0.001 (Ob) vs 0.018 ± 0.001 (Controls), p=575	OR (95% CI)<br>GPX4 SNP rs757228: 0.729 (0.533-0.997), p=0.048<br>GPX4 SNP rs8103188: 0.696 (0.506-0.957), p=0.026<br>GPX5 rs445870: 1.452 (1.043-2.020), p=0.027<br>GPX6 rs406113: 1.562 (1.117-2.184), p=0.009	Adjusted by sex and age under an additive model of inheritance	[73]
Health survey evaluation in Brazil	Trial	2011	37	F	32.4±6.5 (Pro/Pro), 36.1±7.0 (Pro/Leu), 36.8±6.8 (Leu/Leu)	Blood	Mean ± SD<br>**Plasma Se (μg/L)**<br>54.0 ± 12.1 (Pro/Pro), 55.2 ± 14.0 (Pro/Leu), 62.7 ± 16.0 (Leu/Leu) (Baseline) vs 126.6 ± 21 (Pro/Pro), 134.4 ± 40.4 (Pro/Leu), 148.3 ± 45.0 (8w supplementation)			[74]
							RBC Se (μg/L)<br>60.8 ± 18.5 (Pro/Pro), 65.0 ± 37.6 (Pro/Leu), 59.7 ± 23.1 (Leu/Leu) (Baseline) vs 200.8 ± 33.1 (Pro/Pro), 207.3 ± 39.5 (Pro/Leu), 220.2 ± 76.0 (8w supplementation)	GPX activity/RBC Se (Baseline)<br>r=0.60, p< 0.01 (Pro/Pro)<br>r=0.44, p=0.117 (Pro/Leu)<br>r=0.62, p=0.261 (Leu/Leu)		
							GPX activity (U/g Hb)<br>38.5 ± 18.0 (Pro/Pro), 33.0 ± 12.4 (Pro/Leu), 31.4 ± 19.6 (Leu/Leu) (Baseline) vs 57.4 ± 21.5<br>(Pro/Pro), 51.7 ± 19.7	GPX activity/RBC Se (8w supplementation)<br>r=0.43, p< 0.05 (Pro/Pro)<br>r=-0.21, p=0.475 (Pro/Leu)<br>r=-0.53, p=0.358 (Leu/Leu)		

							(Pro/Leu), 45.2 ± 19.1 (8w supplementation)			

MS – metabolic syndrome; Ob – obesity; BMI – body mass index; SDS-BMI – standard deviation scores of BMI; WHR – waist-to-hip ratio; WC – waist circumference; RBC – red blood cells; SNP - single-nucleotide polymorphism; HOMA-IR – homeostasis model assessment-insulin resistance; QUICKI index – quantitative insulin-sensitivity check index; GPX – glutathione peroxidase; SOD - superoxide dismutase; Hb – hemoglobin; TAG – triacylglycerol; HDL – high-density lipoprotein; TC – total cholesterol; MDA – malondialdehyde; Pro/Pro, Pro/Leu, Leu/Leu – GPx1 Pro198Leu polymorphism
