# Supplementary material for: Selenium and Selenoproteins in Adipose Tissue Physiology and Obesity
Source: Biomolecules. 2020 Apr 24;10(4):658. doi: 10.3390/biom10040658 (PMC7225961; doi:10.3390/biom10040658)
Supplement: Supplementary file 1 [file biomolecules-10-00658-s001.zip › Table_S3.pdf]

Table S3. A brief summary of studies demonstrating the association between selenoprotein P (SELENOP) levels and obesity

Population	Study design	Year	N (M/F)	Sex	Age	Specimen	Se levels	Statistics	Adjustments factors	Ref.
Health survey evaluation in Germany	Cross-sectional	2005-2012	905	Both (M/F)	59.4 (57.8-61.1) (Q1) - 61.6 (59.9-63.2) (Q4)	Blood	Mean (95% CI) SELENOP (μg/mL) 2.86 (1.96-3.70) (Q1), 4.52 (3.87-5.98) (Q2), 6.05 (5.32-8.47) (Q3), 11.72 (8.07-15.79) (Q4) BMI: 29.0 (28.3-29.6) (Q1), 27.4 (26.8-28.0) (Q2), 27.5 (26.9-28.1) (Q3), 25.7 (25.1-26.4) (Q4)	ORs (95%CI) for Q1-Q4 MS: 1.62 (1.08-2.43) (Q1), referent (Q2), 0.85 (0.57-1.26) (Q3), 0.41 (0.27-0.62) (Q4) NAFLD: 1.32 (0.79-2.21), referent (Q2), 0.69 (0.41-1.16) (Q3), 0.90 (0.53-1.51) (Q4) Obesity: OR: 0.73; 95% CI: 0.55-0.94	Adjusted for age, sex, physical activity, education, smoking status, alcohol consumption, and C-reactive protein. NAFLD additionally adjusted for prevalent diabetes and BMI.	[76]
Selenegene study	Cross-sectional	2015-2016	131 (99/32)	Both (M/F)	55.9±7.52 (Non-MS), 55.6±6.41 (MS)	Blood	Mean ± SD SELENOP (ng/ml) 41.8±6.57 (MS) vs 81.5±15.2 (Non-MS), p=0.022	OR (95% CI) MS: 0.995 (0.990-1.000)	Adjusted for age, gender, family history and smoking	[76]
Health survey evaluation in Germany	Cross-sectional	2005-2015	553	Both (M/F)	60.6 (59.8–61.5)	Blood	Median (IQR) SELENOP, μg/mL 4.86 (3.30–6.96) (MS) vs 6.15 (4.50–9.55) (Non-MS)	β (95% CI) MS: 0.54 (0.40–0.73) DM2: 0.47 (0.28–0.78) NAFLD: 0.93 (0.65–1.34)	Adjusted for age and sex, physical activity, educational level, smoking status, C-reactive protein, selenium supplementation, total energy intake (for NAFLD + for prevalent diabetes and body mass index	[80]
Korean Metabolic Disorders and Obesity Study	Cross-sectional	2006-2010	210 (113/97)	Both (M/F)	9	Blood	Mean ± SD SELENOP (ng ml^{-1b}) 16.7±2.2 (MS) vs 28.6±2.0 (Non-MS), p <0.001	ORs (95%CI) for T1-T3 MS: 1.0, 0.47 (0.24–0.92), 0.18 (0.09–0.37), p<0.001	Unadjusted	[78]

							SELENOP (Tercile)<br>1.34–16.55 (T1),<br>16.74–32.03 (T2),<br>32.18–80.00 (T3)	MS: 1.0, 0.25 (0.04–1.60), 0.05 (0.00–0.96), p=0.042	Adjusted for sex, physical activity, sleep duration, paternal education level, high-sensitivity C-reactive protein, HOMA-IR and ALT	
Health survey evaluation in South Korea	Follow-up	2016	10<br>(4/6)	Both (M/F)	25–46	Blood	Mean ± SD<br>ln SELENOP, μg/mL<br>1.47 ± 0.11 (Before) vs 1.76 ± 0.10 (1m) vs 2.10 ± 0.08 (9m)	r=−0.770, p<0.05 (Δ HOMA-IR)		[79]
KSOS Study	Prospective observational cohort study	2007-2009	120<br>(59/61)	Both (M/F)	47.0±13.0 (C), 49.1±13.1 (NAFLD)	Blood	Median (IQR)<br>SELENOP, ng/mL<br>530.4 (246.2-1478.2) (Controls) vs 1509.3 (899.0-2773.2) (NAFLD), p<0.001	ORs (95%CI) for T1-T3<br>NAFLD: 1.00, 6.3 (1.51-26.28), p=0.012, 7.48 (1.72-32.60), p=0.007	Adjusted for age, sex, BMI, smoking status, SBP, DBP, triglycerides, HDL-C, high sensitivity C-reactive protein, adiponectin, and homeostasis model assessment of insulin resistance values.	[81]
Health survey evaluation in Australia	Case-control<br>Controls n=29,<br>Ob n=34	2011-2015	63<br>(19/44)	Both (M/F)	23.2±15.5	Blood	Mean ± SD<br>SELENOP, μg/mL<br>14.5 ±12.8 (Controls) vs 52.3 ±39.1 (Ob), p=0.001	BMI: r = 0.55, P< 0.0001<br>HOMA-IR: r = 0.37, p= 0.005<br>TC: r = 0.46, p= 0.001<br>LDL cholesterol: r = 0.35, p= 0.02)<br>TAG: r = 0.48, p= 0.001	Adjusted for sex and age	[82]
							SELENOP1 mRNA expression<br>1.1 ±0.2 (Controls) vs 1.0 ±0.3, p=0.15	BMI: r = −0.44, P= 0.03<br>HOMA-IR: r = −0.46, p= 0.02		
Health survey evaluation in UK	Case-control	2017	22	M	36±15 (C),<br>45±14 (Ob)	Blood	Mean ± SD<br>SELENOP, μg/mL<br>3.01 ± 0.39 (Controls) vs 2.81 ± 0.30 (Ob)			[83]

	Controls n=11, Ob n=11						SELENOP AUC 20.9 (Control) vs 21.0 (Control+Exr) vs 19.8 (Ob) vs 19.5 (Ob+Exr), $p>0.05$			

MS – metabolic syndrome; Ob – obesity; NAFLD – non-alcoholic fatty liver disease; BMI – body mass index; HOMA-IR – homeostasis model assessment-insulin resistance; SELENOP – selenoprotein P; LDL – low-density lipoprotein; HDL – high-density lipoprotein; TAG – triacylglycerol; TC – total cholesterol; ALT – alanine aminotransferase; Exr – exercise; SBP – systolic blood pressure; DBP – diastolic blood pressure
