# Supplementary figures and images for: Selenium and Selenoproteins in Adipose Tissue Physiology and Obesity
Source: Biomolecules. 2020 Apr 24;10(4):658. doi: 10.3390/biom10040658 (PMC7225961; doi:10.3390/biom10040658)

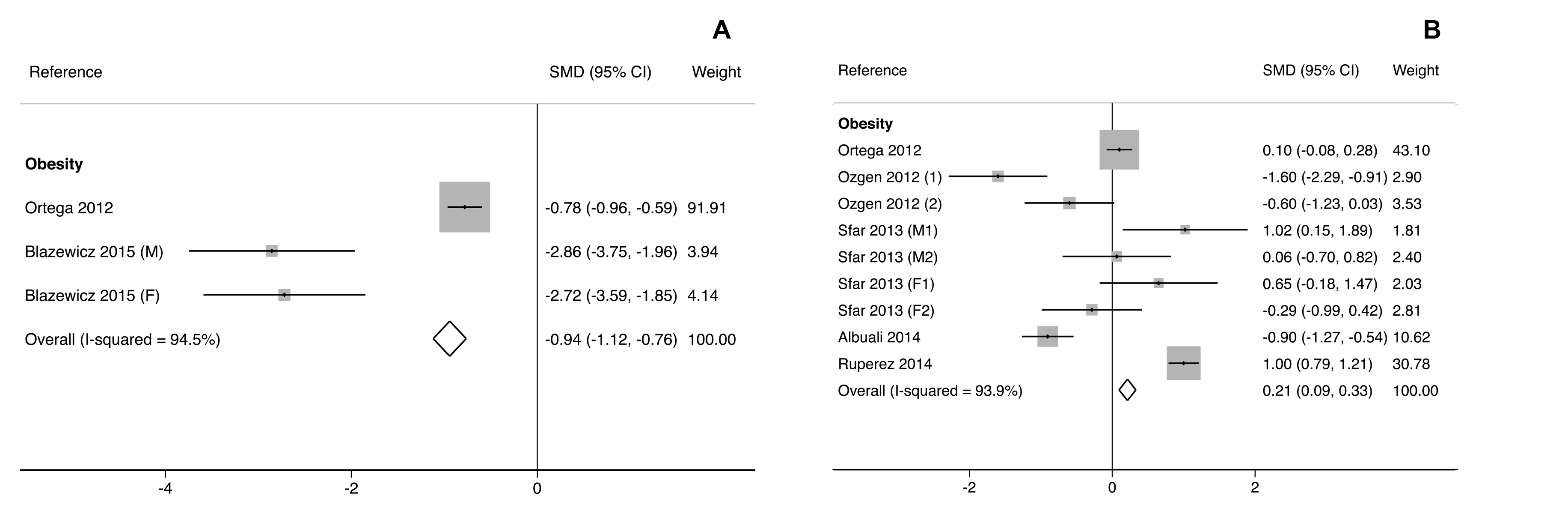

Supplement: Supplementary file 1 [file biomolecules-10-00658-s001.zip › Fig S1.tif]
